# An epidemic model for SARS-CoV-2 with self-adaptive containment measures

**DOI:** 10.1371/journal.pone.0272009

**Published:** 2022-07-25

**Authors:** Sabina Marchetti, Alessandro Borin, Francesco Paolo Conteduca, Giuseppe Ilardi, Giorgio Guzzetta, Piero Poletti, Patrizio Pezzotti, Antonino Bella, Paola Stefanelli, Flavia Riccardo, Stefano Merler, Andrea Brandolini, Silvio Brusaferro

**Affiliations:** 1 Directorate General for Economics, Statistics and Research, Bank of Italy, Rome, Italy; 2 Center for Health Emergencies, Bruno Kessler Foundation (FBK), Trento, Italy; 3 Department of Infectious Diseases, Italian National Institute of Health (Istituto Superiore di Sanità), Rome, Italy; 4 Italian National Institute of Health (Istituto Superiore di Sanità), Rome, Italy; INSERM, FRANCE

## Abstract

During the COVID-19 pandemic, several countries have resorted to self-adaptive mechanisms that tailor non-pharmaceutical interventions to local epidemiological and health care indicators. These mechanisms reinforce the mutual influence between containment measures and the evolution of the epidemic. To account for such interplay, we develop an epidemiological model that embeds an algorithm mimicking the self-adaptive policy mechanism effective in Italy between November 2020 and March 2022. This extension is key to tracking the historical evolution of health outcomes and restrictions in Italy. Focusing on the epidemic wave that started in mid-2021 after the diffusion of Delta, we compare the functioning of alternative mechanisms to show how the policy framework may affect the trade-off between health outcomes and the restrictiveness of mitigation measures. Mechanisms based on the reproduction number are generally highly responsive to early signs of a surging wave but entail severe restrictions. The emerging trade-off varies considerably depending on specific conditions (e.g., vaccination coverage), with less-reactive mechanisms (e.g., those based on occupancy rates) becoming more appealing in favorable contexts.

## Introduction

During the COVID-19 pandemic, public authorities faced the demanding task of adopting mitigation policies to minimize the strain on health systems while considering the implied socioeconomic costs. At the onset of the pandemic in the spring of 2020, several countries resorted to nationwide lockdowns, given the high uncertainty about the impact of the novel disease on the national health systems. These policies had profound effects on both public health [1–6] and socioeconomic variables worldwide [7–15]. After the initial emergency, a new surge in cases in autumn 2020 prompted many countries to introduce tier systems of contingent measures based on epidemiological indicators such as incidence and growth rates of confirmed cases and hospital bed occupancy rates (e.g., Italy [16], Germany [17], the United Kingdom [18], and France [19]). Unlike the initial lockdowns, these mechanisms allow for a dynamic adaptation of non-pharmaceutical interventions (NPIs) to the evolution of the epidemic. Compared with purely discretionary measures, such rule-based systems offer several advantages, including predictability and time-consistency of the responses and geographical differentiation of the interventions within a common nationwide approach.

Analyses, often supported by an underlying epidemiological model, generally, focus on the role of specific policy interventions (e.g., border closure [20], mask mandate [21, 22], remote working [23], vaccination [24], school closures [25–28]). These frameworks assess how introducing a particular policy impacts the epidemic’s evolution, holding everything else fixed. While still valid, such an approach overlooks the critical interplay between the epidemic trajectory and response mitigation policies observed in the real world. In other words, governments tend to calibrate their policies and containment measures depending on the epidemiological situation and its evolution following the enforced interventions. This aspect becomes even more relevant with rule-based mechanisms in which the epidemic and restrictions interact almost automatically. Adequate models are critical to evaluating possible epidemic trajectories under different self-adaptive, reactive mechanisms and informing the decision process of a policymaker aiming to reach her targets in terms of health and social and economic activities.

### Related work and contribution of the paper

A thorough understanding of the relationships between epidemic dynamics, non-pharmaceutical interventions, health outcomes, and economic activity proved crucial for policymakers to assess the impact of the epidemic and of the related policy responses. In some cases, fully-fledged macroeconomic tools have been developed for investigating the effects of pandemic-related outcomes on macroeconomic indicators (e.g., [29–32]) or exploring the spillovers on trade and production networks [33]. However, in previous contributions, containment policies are often modeled in a stylized, simplified manner. This feature may limit the possibility of obtaining a reliable quantification of the effect of NPIs on the epidemic and economic outcomes.

Our paper contributes to the literature on epidemic-policy models, enabling a realistic interplay between containment measures and the evolution of the epidemic. The interplay allows assessing the impact of alternative NPIs mechanisms both on health outcomes and restrictions on social interactions, which are directly related to the economic activity [34–37]. In detail, we integrate an extended SIR model with an algorithmic component enhancing reactive adjustment of the infection rate levels based on epidemic outcomes. Remarkably, the embedded algorithmic component mimics the actual policy mechanism adopted by the Italian government. Weekly rule-based evaluation of epidemiological indicators enforces adaptive regional NPIs, to balance the health-wealth trade-off posed by the COVID-19 pandemic [38]. Then, we test how different policy mechanisms, based on alternative epidemic indicators, influence the health outcomes and restrictions evolution.

To derive the epidemiological indicators required for the functioning of the policy mechanism, we extend the SIR model and account for several transition paths of individuals across compartments, allowing for geographic and demographic heterogeneity of the Italian population.

Other works by national and international policy institutions featured a static policy-oriented component and a SIR model to address such a task [39–41]. To the best of our knowledge, our contribution is the first to model the interplay between policy interventions and epidemic dynamics in a fully-fledged, realistic framework.

## Materials and methods

### Model

**Fig 1:**
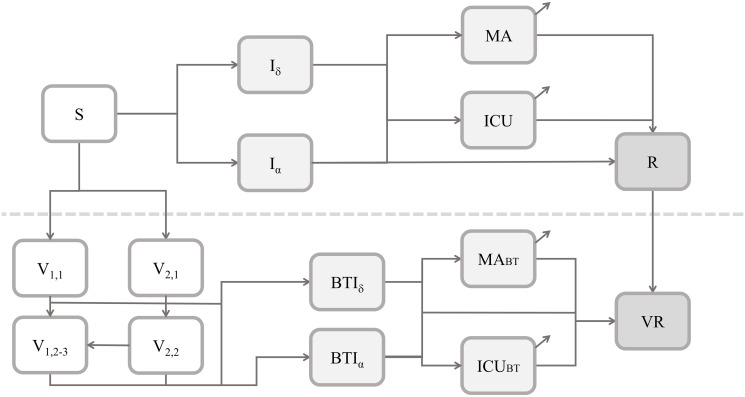
Representation of the epidemiological compartmental model. Model compartments: Susceptible to the virus (S), infectious with Delta (I_ζ_) and with Alpha (I_α_), hospitalized in the medical area (MA) and intensive care unit (ICU), recovered from natural infection (R), immunized with the first group of vaccines (first and second/third dose, respectively, V_1,1_ and V_1,2−3_) and with the second group of vaccines (first and second dose, respectively, V_2,1_, V_2,2_), breakthrough infectious with Delta (BTI_ζ_) and Alpha (BTI_α_), hospitalized with breakthrough disease (MA_BT_, ICU_BT_) and recovered from breakthrough infection (VR). The dashed line separates the dynamics associated with natural infections from those of vaccinated individuals. Each compartment is stratified by age class and region. See S1 Appendix for details.

The core of our framework is the interaction between the SARS-CoV-2 transmission and restrictions. We model the transmission for each Italian region and autonomous province with an age-structured compartmental model, which extends the workhorse SIR framework [42] by accounting for different courses of the symptomatic disease, variants of the virus, effects of temperature, types of vaccines, and progress in the vaccination campaign (Fig 1). Regarding vaccines, we incorporate the currently available evidence regarding their waning immunity against infection and protection against severe disease [43–45].

Our model mimics the response mechanisms adopted by the Italian Ministry of Health (MoH) between November 2020 and March 2022. Regional epidemiological variables define weekly indicators (e.g., case incidence, reproduction number, hospital occupancy rates), which constitute the inputs of an algorithm producing restriction tiers and the related containment policies for the ensuing week. The enforcement of diverse degrees of restrictions characterizes heterogeneously the evolution of the epidemic and the corresponding indicators across regions, affecting the path of future restrictive measures (see S1 and S2 Appendices) [46].

We stratify the Italian population by geography and age. Concerning the former, we follow the Nomenclature of Territorial Units for Statistics—Level 2 (NUTS 2), dividing the Italian territory into 19 regions and two autonomous provinces. In line with this, we introduce region-specific fixed effects in infection transmission to reflect regional heterogeneity in characteristics that may influence the contagion (e.g., population density, climate and pollution conditions, and individual behaviors). Regarding the age classification, we consider five mutually exclusive groups: 0–12, 13–18, 19–64, 65–79, and 80+. Our model does not incorporate movement across regional borders and transition between age groups.

Besides the above stratification, we augment the model with additional compartments for different courses of the symptomatic disease, vaccine types, and virus variants. Finally, we allow for a time-varying parametrization reflecting changes in the seasonal conditions, coverage, and efficacy of the vaccines by region and targeting group.

We account for the presence of strains of SARS-CoV-2 with different characteristics. The evidence available through the Italian National Institute of Health (Istituto Superiore di Sanità, henceforth ISS) shows that two variants were highly prevalent in the country in the two halves of 2021: *Alpha* until June, and *Delta*, from July onward [47, 48]. We parameterize the features of each according to the available literature, showing that Delta is significantly more transmissible than Alpha [49] and twice as likely to result in hospitalization among non-vaccinated individuals [50].

In December, a new variant, *Omicron*, spread in the country [51]. Although the simulations cover July 2021—March 2022, we do not account for Omicron when calibrating the relevant parameters, and we abstract from its impact on the epidemic situation. This simplification allows us to appreciate more clearly the policy mechanisms’ functioning under heterogeneous conditions. Ideally, one wants to evaluate a mechanism by considering an entire epidemic wave to observe the full path of health and restriction outcomes and construct the relevant indicators for the analysis. Such an approach reduces the likelihood of a misjudgment induced by the use of partial information. For this reason, we do not limit the simulation when Delta prevailed, and daily cases were still rising (i.e., July—December 2021). Similarly, we neglect the insurgence of Omicron (December 2021) in the simulations as it would add a source of uncertainty to the model’s parametrization and complicate the interpretation of the results. The period December 2021—March 2022, in which Omicron spread in the country, is not used for calibrating the relevant parameters (see Calibration). Nonetheless, we test that results are qualitatively unchanged when including Omicron in the model.

### Vaccination campaign

During the simulation period, the vaccination campaign in Italy has relied mainly on four approved vaccines: two mRNA vaccines (Comirnaty by Pfizer/BioNTech and Spikevax by Moderna) and two viral vector vaccines (Vaxzevria by Oxford University and AstraZeneca and the COVID-19 Vaccine by Janssen/Johnson&Johnson). For simplicity, we group them into two classes: 1) Comirnaty/Spikevax and 2) Vaxzevria/COVID-19 Vaccine Janssen. The two classes differ in the administration mode and efficacy in preventing infections, hospitalizations, and deaths. In addition, we differentiate the protection against the disease among immunized individuals according to the number of doses received—first, second, and booster—and variant type since vaccines appear less effective against Delta than Alpha [52]. Regarding Delta, vaccines are still protective among fully vaccinated individuals [53] and can prevent hospital admissions in most cases [50]. The available evidence also shows that the protection associated with vaccination wanes over time. We account for this finding by accounting for the age-dependent, vaccine-specific reduction in protection against infection and risk of hospitalization over time. Alongside effectiveness, we track differences in vaccination uptakes across regions and age groups by using historical data until November 2021, available through the GitHub repository maintained by the Italian Civil Protection [54]. The data contain aggregate information on vaccine rollout by date, region, age group, and vaccine type, allowing us to compute the *age* of each vaccinee cohort for each region and vaccine type. Such information is then used with the available evidence on waning immunity at different time horizons to obtain the average vaccine efficacy used in the model for each age group, vaccine cohort, region, and vaccine type.

We consider three scenarios for the vaccination campaign. First, *Actual rollout* relies on historical data. Second, *Optimistic rollout* assumes a faster rollout than observed between June and November (20% faster in the second and third age groups, 55% for the fourth age group, and 105% for the fifth age group). Third, *Pessimistic rollout* assumes a slower rollout than observed (80% of actual doses for all age groups between June and December).

For December 2021—March 2022, we consider a reprise of first doses in all scenarios, which should fit the increase due to the extended application of COVID-19 certificates. However, the slowdown of new first doses differs across scenarios, with *Pessimistic* (*Optimistic*) *rollout* showing a faster (slower) reduction of first doses than *Actual rollout*. Regarding boosters, we replicate the policy enacted by the Italian government in *Actual rollout*. In September, boosters were available to the elderly six months after the second dose [55]. Later, the Italian government extended the criteria for eligibility to younger cohorts and shortened the minimum distance between the second and third doses to five months [55] and finally to four [55]. *Optimistic* (*Pessimistic*) *rollout* assumes a fast (slow) rollout of boosters to eligible individuals. Moreover, vaccinated individuals may access boosters four months after full vaccination in *Optimistic rollout*. Since we do not have individual-level information on the time span between the vaccinations, the allocation of boosters in our simulations works according to a first-in-first-out principle.

### Policy mechanisms

We focus on four alternative policy-response mechanisms, three enforced in Italy after November 2020. The first one (*Rt-New Positives*), in place from March 2021 to May 2021, is mostly based on the reproduction number, *Rt*—i.e., the expected number of secondary cases per infectious individual at a given time *t*—estimated on reported symptomatic cases and the weekly incidence [56]. The latter is primarily used by the second mechanism (*Incidence*), in force between May—July 2021. The third mechanism (*Occupancy rates*), effective between July 2021—March 2022, considers occupancy rates of non-critical medical area (MA) and intensive care unit (ICU) beds as leading indicators [46]. On top of the implemented schemes, we also design an additional mechanism that might be interesting from a policy perspective. The mechanism takes the reproduction number estimated on hospital admissions in MA (*Rt-Hospital admissions*) as the leading policy indicator. Compared to *Rt-New positives*, this alternative scheme may accommodate the decoupling between the evolution of new cases and hospital admissions induced by the high and long-lasting vaccine protection against severe disease. Furthermore, the possibility of introducing a newly designed mechanism proves the flexibility of our approach.

Following the weekly collection of epidemic indicators carried out by the Italian MoH, the policy mechanism defines the tier-based restrictions through an algorithm depending on the value of the indicators themselves. In particular, the MoH assigns each Italian region to a zone with increasingly restrictive containment measures—white (mild restrictions), yellow, orange, or red (near-lockdown provisions). Although the Italian government adjusted the rule-based mechanism over time to accommodate changes in the epidemiological setting (e.g., the onset of new variants, the progressive achievement of high vaccination coverage), the provisions within each tier have been mostly consistent over time, at least for unvaccinated individuals. The four mechanisms are evaluated in terms of critical epidemiological indicators, like the number of daily new cases, occupancy rates in MAs and ICUs, and the Italian Stringency Index (ItSI) [46], an *ad-hoc* synthetic indicator measuring the intensity of restrictions implemented in Italy throughout the pandemic adapted from the Oxford Stringency Index [57].

### Calibration

Model parameters are adopted from published estimates or directly estimated from ISS COVID-19 Surveillance epidemic data [58], except for two sets of free model parameters for each considered region (S1 Table).

The first set of parameters represents the relative regional effectiveness of tier provisions. These parameters are scale factors for transmissibility that reflect local dynamics relatively to a national baseline (see S1 Appendix for details). Since the complex nature of our developed framework prevents analytical formulation of the optimization problem, the value for each region is optimized via grid search (with step size *ϵ* = 1*e* − 4) to minimize empirically the mean squared error between actual and model-based incidence for the period November 9—December 30, 2020. The range of the uniform grid is identified from the relation between the regional reproduction number and observed policy tiers focusing on the periods before and after the introduction of the tier-based policy mechanism (October 1, 2020—January 25, 2021) (see S1 Appendix). For this purpose, we rely on the observed tier restrictions, excluding the algorithm that defines policy tiers in a self-adaptive way from the model. Once the optimal regional values are derived, we restore the algorithmic mechanism and let restrictions be determined endogenously within our model for validation. Then, we verify that the model accurately tracks both the epidemic conditions and the restrictions (S1 Fig).

The second set of parameters is the regional prevalence of Delta on June 14, 2021. We calibrate prevalence levels by using a grid search with step size *ϵ* in the unit interval to minimize collectively the mean squared error between the actual and model-based incidence over three weeks (June 14—July 4, 2021), taking the restrictions as given.

## Results

This section shows how our approach may provide illustrative scenarios for the evolution of the COVID-19 epidemic. We assess and compare the impact of different mechanisms of self-adaptive interventions on health and social-interaction outcomes. Moreover, we show how other external policy-relevant variables (e.g., vaccination rollout and uptake) may influence the effectiveness of various self-adaptive mechanisms.

### Comparison among self-adaptive mechanisms

**Fig 2:**
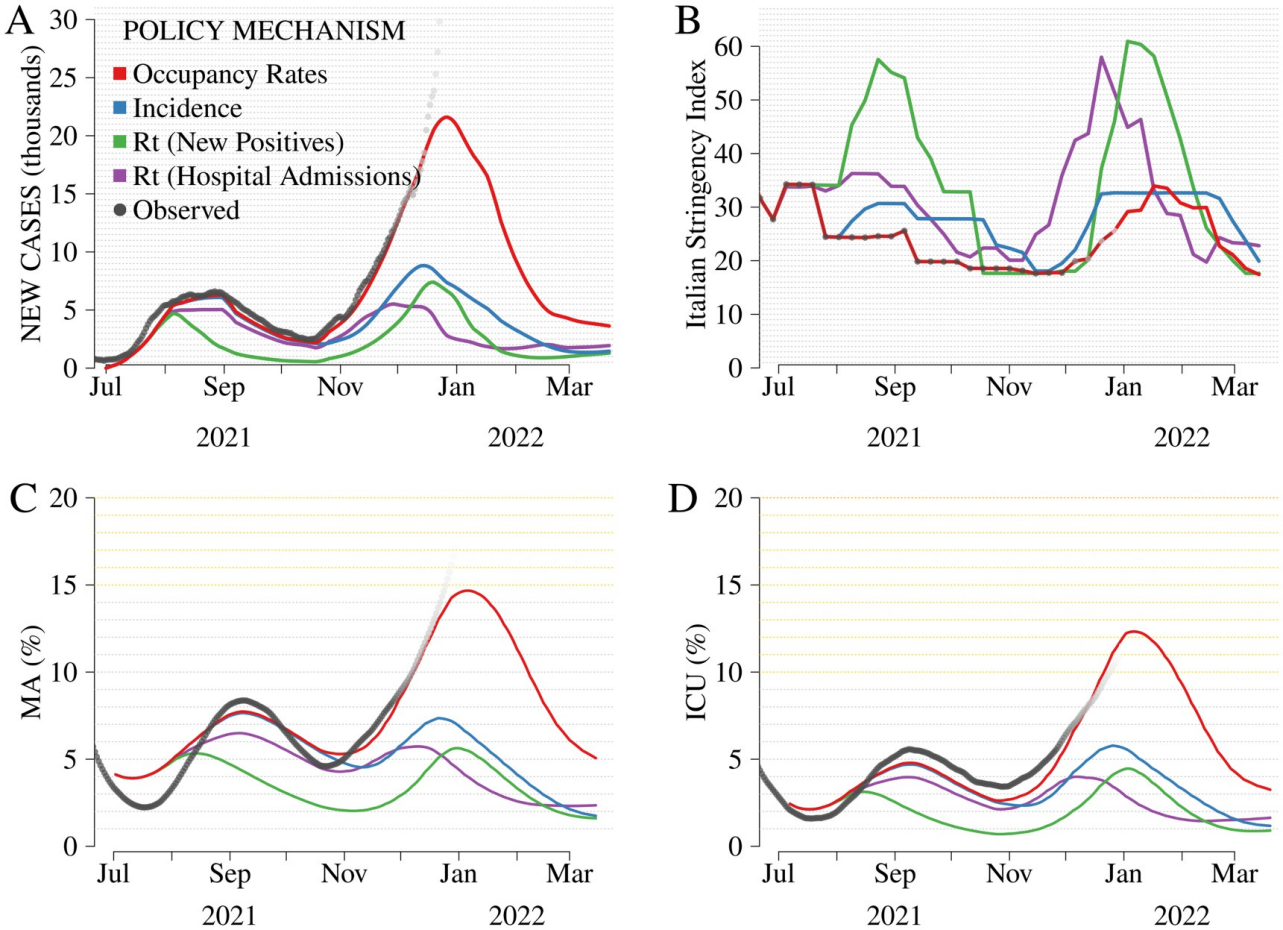
Comparison among mechanisms, *Actual rollout*. From left to right and top to bottom: new cases (thousands, Panel A), Italian Stringency Index (ItSI, Panel B) [46], occupancy rates in non-critical medical areas (MA, Panel C), and intensive care units (ICU, Panel D). Gray dots represent observed data (increasingly shaded to highlight the decoupling between model assumption and the actual unfolding of the pandemic due to Omicron). The mechanisms evaluated are i) *Occupancy rates*, reproducing the mechanism in force in Italy from July 2021 to March 2022, ii) *Incidence*, reproducing the mechanism in force from May to July 2021, and iii) *Rt (New Positives)*, reproducing the mechanism in force from March to May 2021, and iv) *Rt (Hospital Admissions)*, a fictitious framework based on *Rt (New Positives)* in which the reproduction number, *Rt*, relies on hospital admissions (instead of new cases). The hypotheses underlying *Actual rollout* are further described in S4 Appendix.

Fig 2 shows the evolution of the target variables obtained assuming the actual evolution of the ongoing vaccination campaign (*Actual rollout*). Two epidemic waves occur under all mechanisms, accurately reflecting the historical data. The summertime wave is attributable to the onset of Delta, whose spread was boosted by the increased mobility observed throughout the first decade of July, coinciding with the European Football Championship [59]. The fall-winter wave was driven by several factors, possibly including the waning efficacy of the vaccines and the increase in indoor social interactions associated with dropping temperatures, school re-openings, and a substantial return to workplaces. However, some relevant differences across the mechanisms emerge (S2 Table).

First, *Occupancy rates* is the one that leads to the highest incidence (Fig 2A), which results in the highest occupancy rates in the hospitals (Fig 2C and 2D). Indeed, under this scheme, containment measures are less responsive to changes in epidemic conditions, which entails longer waves of infections. At the same time, this low responsiveness implies a material reduction in the restrictiveness indicator as compared to the other policy frameworks (Fig 2B).

Second, *Rt-New positives* guarantees consistently fewer infections and hospitalizations than the other mechanisms but at the cost of stricter containment measures. By relying on the effective reproduction number of detected cases, the mechanism determines a swift increase in restrictions (Fig 2B) during the summer wave (Fig 2A). However, the low incidence achieved at the beginning of the fall, combined with the progress in the vaccination campaign, allows for a delayed wave and a low level of restrictions until November-December 2021. Restrictions increase steeply at the beginning of 2022 when the epidemic also accelerates under this mechanism. Compared to *Occupancy rates*, *Rt-New positives* reduces reported cases by about 4,800 and hospital bed occupancy rates in non-critical medical areas by 4.2 percentage points (p.p.) and 3.4 p.p. in ICU over the entire simulation period. At the same time, the ItSI is about 40% higher, which implies a considerable increase in the socioeconomic costs related to social distancing provisions.

*Incidence* and *Rt-Hospital admissions* occupy intermediate positions in the health-stringency spectrum. The two mechanisms are associated with similar cases and hospital admissions, and the overall level of restrictions is comparable until fall 2021, albeit with different timing. *Rt-Hospital admissions* tends to react more promptly than *Incidence* to a change in epidemic conditions. Noticeably, the progress in the vaccination campaign achieved by the end of the year determines that the hospital bed occupancy rates associated with *Rt-Hospital admissions* and *Incidence* are close. However, unlike the summer wave, the former triggers substantially stricter restrictions than the latter.

### Role of vaccination

The evaluation of different mechanisms may also depend on external conditions regarding, for instance, the virus’s characteristics, the vaccination campaign’s progress, vaccine efficacy, and waning protection. To assess the impact of external conditions on the interplay between health and policy outcomes, we focus on the vaccination campaign. Since their approval and initial distribution, vaccines have been a critical policy variable in tackling the COVID-19 pandemic for their ability to shape the relationship between restrictions and epidemic outcomes [60, 61]. For example, low vaccine protection or coverage may make adopting highly responsive mechanisms desirable to limit the burden of a massive epidemic outbreak on the national health system.

**Fig 3:**
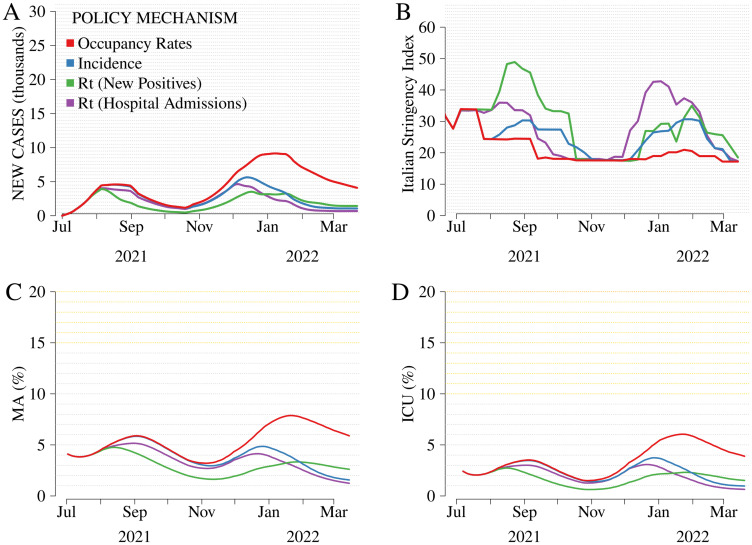
Comparison among mechanisms, *Optimistic rollout*. From left to right and top to bottom: new cases (thousands, Panel A), Italian Stringency Index (ItSI, Panel B) [46], occupancy rates in non-critical medical areas (MA, Panel C), and intensive care units (ICU, Panel D). The mechanisms evaluated are i) *Occupancy rates*, reproducing the mechanism in force in Italy from July 2021 to March 2022, ii) *Incidence*, reproducing the mechanism in force from May to July 2021, and iii) *Rt (New Positives)*, reproducing the mechanism in force from March to May 2021, and iv) *Rt (Hospital Admissions)*, a fictitious framework based on *Rt (New Positives)* in which the reproduction number, *Rt*, relies on hospital admissions (instead of new cases). The hypotheses underlying *Optimistic rollout* are further described in S4 Appendix.

**Fig 4:**
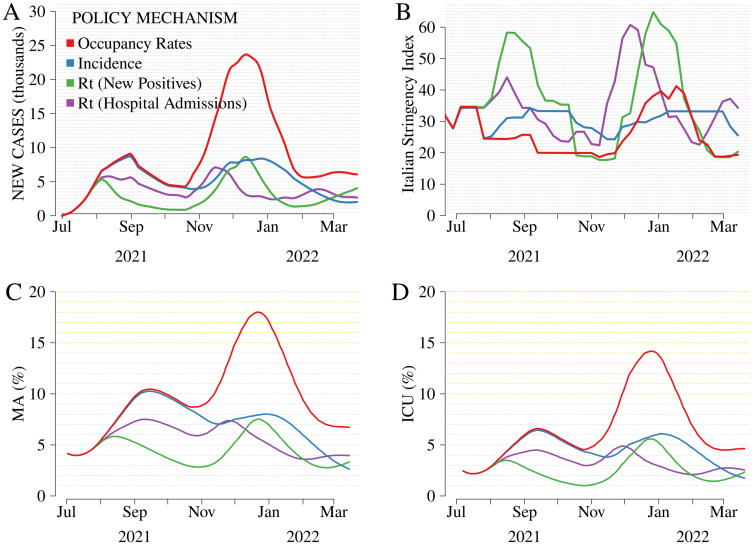
Comparison among mechanisms, *Pessimistic rollout*. From left to right and top to bottom: new cases (thousands, Panel A), Italian Stringency Index (ItSI, Panel B) [46], occupancy rates in non-critical medical areas (MA, Panel C), and intensive care units (ICU, Panel D). The mechanisms evaluated are i) *Occupancy rates*, reproducing the mechanism in force in Italy from July 2021 to March 2022, ii) *Incidence*, reproducing the mechanism in force from May to July 2021, and iii) *Rt (New Positives)*, reproducing the mechanism in force from March to May 2021, and iv) *Rt (Hospital Admissions)*, a fictitious framework based on *Rt (New Positives)* in which the reproduction number, *Rt*, relies on hospital admissions (instead of new cases). The hypotheses underlying *Pessimistic rollout* are further described in S4 Appendix.

We conduct simulations assuming higher and lower exogenous vaccine coverage than the baseline (*Actual rollout*). We label these simulations as *Optimistic rollout* and *Pessimistic rollout*, respectively. *Optimistic rollout* (Fig 3) assumes a faster rollout, a larger final uptake among the population, and a faster deployment of the third dose (booster). Conversely, *Pessimistic rollout* (Fig 4) relies on a slower and less extensive rollout than observed. All simulations account for uncertainty in the vaccination-induced immunity (S4 Appendix for more details).

Results confirm that vaccination coverage is a crucial variable interacting with the policy mechanisms. In the counterfactual *Optimistic* (*Pessimistic*) *rollout* scenario, new cases, occupancy rates of hospital beds, and the ItSI would all reach remarkably lower (higher) levels than *Actual rollout*.

In *Optimistic rollout*, vaccines effectively slow down the spread of the disease in the population, reduce the share of infected requiring medical treatment, and drag down the level of restrictions needed to contain the epidemic by acting effectively on the respective underlying indicator. For instance, average MA occupancy rates are 0.5–1.9 p.p. lower in *Optimistic rollout* than in *Actual rollout*, with the difference varying across mechanisms (S2 Table). Indeed, *Optimistic rollout* is characterized by a lower level of heterogeneity in cases and hospital admissions across the mechanisms. *Rt-New positives* is still associated with the lowest number of daily newly reported cases (2,074 on average throughout the considered period). However, the gains from decreased hospitalizations are considerably reduced relatively to *Actual rollout*. Conversely, under these favorable conditions, health outcomes under *Occupancy Rates* are closer to those produced by the other mechanisms with a material reduction of restrictions. The difference in MA occupancy rates between *Occupancy Rates* and *Rt-New positives* is about 2.2 p.p. on average (1.8 for ICU), while the ItSI remains about 40% higher under *Rt-New positives*. This evidence suggests that a policymaker aiming to reduce restrictions may prefer a mild policy framework, such as *Occupancy Rates*, when external conditions are propitious (e.g., high vaccination coverage or low transmissibility of the virus).

Findings are much more heterogeneous under *Pessimistic rollout*. In this case, *Occupancy Rates* leads to a substantial increase in hospitalizations and more impactful and persistent waves than the other mechanisms. Restrictions are still lower during the summer when climatic conditions reduce transmissibility but tend to increase substantially in the fall-winter wave. *Occupancy rates* determines a high ItSI, larger than that implied by *Incidence* during the same wave. Restrictions under *Occupancy rates* are not far from that reached with other mechanisms (-15.3% compared with *Incidence*, -28.6% compared with *Rt-New Positives*; S2 Table), but the delayed response leads to hospital occupancy rates that are about twice as large as those obtained with the other mechanisms. More responsive schemes, e.g., those relying on reproduction numbers, materially reduce hospital admissions in this unfavorable scenario. On average, restrictions associated with responsive mechanisms are large, but the differences from the other mechanisms are smaller than those found in *Actual rollout* and *Pessimistic rollout*. In this contest, *Rt-Hospital admissions* seems a valuable alternative to *Rt-New positives* in force in Italy throughout the spring of 2021. While the restrictions of the two mechanisms are close during the winter wave, *Rt-Hospital admissions* is much more lenient than *Rt-New positives* during the previous summer wave.

**Fig 5:**
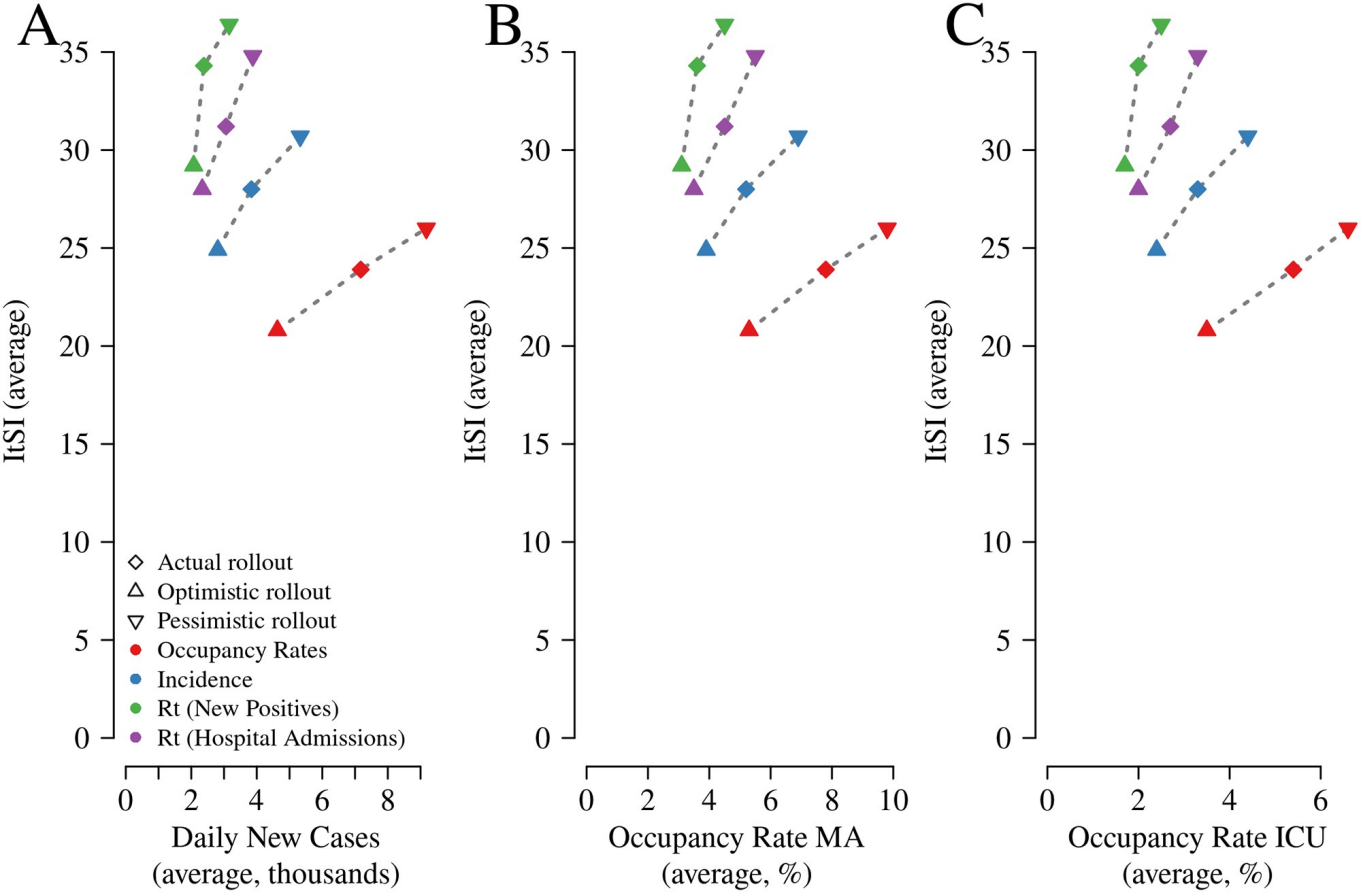
Trade-off between average restriction levels, as measured by the ItSI, and the average values of daily reported new positives (panel A), MA occupancy rate (panel B), ICU occupancy rate (panel C) under each considered vaccine rollout (*Actual rollout*, *Optimistic rollout*, and *Pessimistic rollout*) and mechanism (*Rt-New positives*, *Incidence*, *Occupancy rates*, *Rt-Hospital admissions*), July, 2021—March, 2022. The diamonds refer to *Actual rollout*, the triangles to *Optimistic rollout*, the nablas to *Pessimistic rollout*. Green symbols refer to *Rt-New positives*, blue symbols to *Incidence*, red symbols to *Occupancy rates*, purple symbols to *Rt-Hospital admissions*. In each panel and for a given vaccine rollout, there is an inverse relation between the level of restrictions, as measured by the ItSI, and the value of the corresponding epidemiological indicator during the considered period.

Fig 5 summarizes the above discussion, highlighting the trade-off between epidemic/health outcomes and restrictions under varying external conditions and policy mechanisms.

### Other applications

Integrating endogenous mitigation responses in epidemic models may prove helpful in various applications beyond the comparison between different policy mechanisms. Taking into account self-adaptive NPIs allows simulation results to track the historical epidemic data much more precisely than models with fixed restrictions over time (S5 Appendix). This feature can also be exploited in projection exercises to investigate the possible future trajectories of epidemic variables and restrictions. Indeed, simulations based on the model presented in this work have been used to incorporate the projections of epidemic variables in forecasting models targeting the growth of Italian gross domestic product [62].

Accounting for the reaction of containment policies is crucial also for counterfactual and *ex-post* evaluations of a given exogenous event. For example, we have discussed the effects of different vaccine rollout, considering the possible restrictions changes (Vaccination campaign). In S6 Appendix, we provide an additional example of possible counterfactual analyses that can be conducted within the proposed modeling framework. In particular, we assess the determinants of the divergence between the epidemics in the United States and the European Union observed during the first half of 2021. Since late February 2021, the United States showed a lower incidence than most EU countries, despite more intense community mobility. The fast rollout in the United States in the first phases of the vaccination campaign may be a possible explanation for this parting. However, another relevant factor may be the prevalence of different variants hitting the two regions: at the time, Alpha, more contagious than the wild-type, was more widespread in the European Union than in the US.

The proposed modeling framework can help disentangle the contribution of each component (vaccination rollout and variant prevalence). In a counterfactual simulation, we apply to Italy the favorable conditions prevailing in the United States regarding vaccination rollout and variant prevalence for the period February—May 2021. A delayed diffusion of the new variants would reduce substantially new cases and the severity of restrictions. A faster vaccine rollout would have further eased restrictions and a drop in cases. Results suggest that postponing the diffusion of these variants through controls on international movements and identification, isolation, and monitoring of new outbreaks can help curb the epidemic. The benefits from such an approach are akin to those of a substantial acceleration in vaccination.

## Discussion

Many countries resorted to geographically differentiated, reactive measures in their fight against COVID-19. This approach offers several advantages compared with discretionary strict nationwide interventions. However, it also requires assessing the adequacy of a policy in which the policymaker commits to a course of actions under rapidly evolving external conditions (e.g., diffusion of new variants of concern, the progress of the vaccine campaign). Dynamic assessment of policy effectiveness is crucial to projecting credible epidemic scenarios and related restrictions over different time horizons. The framework presented in this paper can help policymakers choose the appropriate criteria to strike a balance between minimizing the health damages induced by the epidemic and the severity of restrictions.

In this paper, we account for the interaction between containment policies and the evolution of the pandemic by embedding rule-based, self-adaptive policy restrictions into an epidemic model and show the insights that such an enriched model brings. Different rules translate into diverse outcomes regarding restrictions and the epidemic. The complex interplay between restrictions and uncertain epidemiological conditions may magnify differences across viable regulatory frameworks.

The extension of the SIR model to include different self-adaptive policy mechanisms presented in this paper replicates the Italian framework in force between November 2020 and March 2022, which assigned regions to restriction tiers depending on specific epidemic indicators. In this manner, we can simulate the evolution of the variables of interest during the diffusion of Delta from July 2021 to March 2022. As NPIs are not the only available tool, we also consider alternative scenarios for the vaccination rollout to provide a spectrum of outcomes that policymakers may face under various external conditions. Introducing a self-adaptive rule-based policy mechanism differs substantially from exploring the set of available policies with a standard SIR model because, in the former case, NPIs are automatically activated and deactivated depending on their effects on the epidemic’s trajectory.

Simulations show that policy mechanisms based on the reproduction number reduce the impact on the health system compared to alternative mechanisms based on stock variables (e.g., hospital bed occupancy rates or incidence), especially with low vaccine coverage among the population. The rationale is that it takes time before the disease spreads enough to raise the occupancy rate or the incidence (i.e., the pivotal epidemiological indicator) to the threshold that triggers restrictions. Likewise, the effects of NPIs require time to show up in the data since some degree of inertia may characterize the evolution of the epidemic. Conversely, responsive mechanisms generally entail stricter containment measures (see also [63] on this trade-off). The trade-off between health outcomes and the intensity of social interactions ensured by the various regimes crucially depends on the external context. With favorable conditions, such as high vaccination coverage, mild policy schemes guarantee a sizable reduction in restrictions with a small increase in cases and hospital admissions. On the other hand, with low levels of vaccination, responsive mechanisms substantially reduce cases and hospitalizations, as restrictions kick in early and substantially. Clearly, further elements (e.g., the availability of effective treatments, the costs associated with long-term sequelae of COVID-19 infections, loss in school days or working hours linked to quarantines) may affect this trade-off. Ultimately, the evaluation of the trade-off depends on the policymaker’s (and public opinion’s) preferences over epidemiological, economic, and social outcomes. By pinning down some key variables, our framework improves the transparency of such an assessment while being flexible enough to include other aspects characterizing the trade-off.

While we consider many possible sources of uncertainty in our simulations, we remain agnostic on some other relevant factors. For example, the lack of robust evidence makes it hard to estimate the effectiveness of individual interventions (e.g., contact tracing, school closures). To get around this limitation, we considered the overall impact of tier-related provisions on the epidemic (rather than individual ones) by including changes in community mobility indicators as a proxy of their effectiveness and calibrating regional elasticities to consider the geographic heterogeneity of such effectiveness. Notification rates are likely to vary over time due to the incidence of infections, testing capacity, and existing policy regulations. Vaccination rates are also likely influenced by the policy framework and the evolution of epidemics and health conditions [64, 65]. Individual behavior may change over time or across locations due to the so-called “lockdown fatigue” [66] or the adaptation to the varying external conditions, including vaccination status and incidence levels. Moreover, the evaluation of costs and benefits associated with restrictions may substantially vary depending on the state of the epidemic itself. With insufficient data to model these effects, quantitative outcomes associated with different epidemiological scenarios should be considered illustrative and valuable to evaluate the potential interplay between the disease spread and endogenous mechanisms to counter an increase in COVID-19 infections.

Due to the already high complexity of the model, we decided to simplify the representation of other relevant aspects of SARS-CoV-2 epidemiology. For example, we did not include mobility between regions [67, 68], and we considered a relatively coarse partition of the Italian territory. We also did not consider the heterogeneity in infectiousness across symptomatic states or the possibility that immunity from natural infection may wane over time [60, 69]. Moreover, our model does not include population dynamics—other than COVID-19-induced mortality—due to their negligible impact over the short time horizon of the simulations.

Finally, although our framework is general enough to fit a large number of rule-based mechanisms, as the proposed fictitious policy and vaccination scenarios show, we do not model the selection of an optimal mechanism because this problem would require, among others, embedding policymakers’ preferences into the set of possible outcomes. There is an important warning in the background of our analysis regarding this choice. Informing rule-based policies requires producing timely and reliable data [70]. Poor information may lead to inadequate measures and jeopardize public trust, which is crucial to containing a pandemic successfully.

Despite these difficulties, including a policy-response algorithmic component in a fully-fledged epidemic model constitutes an essential step forward in designing evidence-informed responses, especially within a rule-based framework. Throughout the COVID-19 pandemic, many valuable contributions have tried to bridge the gap between epidemiology and economics [36, 37, 71, 72]. Including containment policies into an epidemiological model is key to designing policies that improve socioeconomic and sanitary outcomes.

## Conclusion

This paper proposes an epidemiological model with self-adaptive dynamic NPIs, which may help evaluate the potential impact of the pandemic and related restrictions. For its realistic setup and ability to track simultaneously the actual evolution of epidemic conditions and related restrictions, our model may have a broad scope for policy applications. For instance, it may provide useful inputs for exercises aiming to forecast the economic activity or conduct cost-benefit analyses. The proposed framework can be easily adapted to evaluate rule-based policies *ex ante* and *ex post* in different countries with appropriate fine-tuning of the parameters and support informed policy choice among alternative response mechanisms.

## Supporting information

S1 Appendix The compartmental population model. (PDF)

S2 Appendix The tier system in Italy. (PDF)

S3 Appendix The Italian Stringency Index. (PDF)

S4 Appendix Vaccine rollout and coverage rate scenarios. (PDF)

S5 Appendix Fixed restrictions vs. self-adaptive rule-based mechanism. (PDF)

S6 Appendix Assessing the “decoupling” between the United States and Italy. (PDF)

S1 Fig Model calibration. (PDF)

S1 Table New positives, MA occupancy rate, ICU occupancy rate, and the ItSI for each mechanism. (PDF)

S2 Table New positives, MA occupancy rate, ICU occupancy rate, and the ItSI for each mechanism. (PDF)
