# Supplementary material for: An epidemic model for SARS-CoV-2 with self-adaptive containment measures
Source: PLoS One. 2022 Jul 25;17(7):e0272009. doi: 10.1371/journal.pone.0272009 (PMC9312378; doi:10.1371/journal.pone.0272009)
Supplement: S1 Appendix — (PDF) [file pone.0272009.s001.pdf]

S1 Appendix. The compartmental population model

The model

This subsection provides details about the epidemiological model. We develop a framework comprising an SIR component with compartments and a reactive policy component that models the different algorithms for the definition of restrictions implemented between November 2020 – March 2022.

Population compartments in the epidemiological model are stratified by geography and age. We use $r = 1, \dots, 21$ to denote Italian regions according to the Nomenclature of Territorial Units for Statistics – Level 2 (we treat the autonomous provinces of Bolzano and Trento as regions). We consider five age groups, a, with $a \in \{0-12, 13-18, 19-64, 65-79, 80+\}$. Time is defined at a daily frequency, with each step denoted by t. Let p denote the policy tier, with $p \in \{$*white, yellow, orange, red*$\}$. Finally, subscript $j = 1, 2$ denotes variant type, $k = 1, 2$ vaccine groups, and $d = 1, 2$ the number of vaccine doses received, respectively.

While we assume the population to be fully susceptible to COVID-19 at the beginning of the pandemic, we initialize state variables to match detailed information on cases, fatalities, and hospitalizations provided by the Italian National Institute of Health (Istituto Superiore di Sanità, ISS) on a regional basis, in combination with estimates on age-specific Infection Fatality Rates (IFRs) drawn from the literature.

For each r and a, the cumulative number of individuals that have already contracted the virus up to day t, $INF_{a,r}(t)$, is estimated as:

$$INF_{a,r}(t) = \sum_{s=0}^{t} \frac{m_{a,r}}{IFR_a} H_{a,r}(s),$$

where $H_{a,r}(s)$ is the number of new hospitalizations that occurred in r at time s, with $s \leq t$; $m_{a,r}$ is the ratio between hospitalizations and fatalities from the ISS COVID-19 surveillance data [1] for a given combination of r and a; IFR_a is the Infection Fatality Rate of a [2].

We compute the notification rate, $\delta_{a,r}(t)$, for a and r by taking the ratio between the cumulative sum of notified cases up to day t for region r and age group a, $CASES_{a,r}(t)$, and estimated infections, $INF_{a,r}(t)$:

$$\delta_{a,r}(t) = \frac{CASES_{a,r}(t)}{INF_{a,r}(t)}$$

To account for changes in testing strategies, we derive the values for the notification rate based on the period ranging from October 1, 2020, to January 18, 2021. Age- and region-specific rates are used to initialize the model and simulate the number of detected cases from actual new infections, with average estimated notification rates equal to 36.0% – 49.1%.

We consider the following model compartments:

- ***S***: *Susceptible individuals* are at risk of being infected by the virus. The per capita rate $\lambda_{a,r,j,p}(t)$ at which individuals acquire the infection is called Force of Infection (FoI), defined for each variant j. The FoI depends on time, variant, age group, region, and ongoing policy, and according to

$$\lambda_{a,r,j,p}(t) = \beta_j \beta_a (1 - \phi_p) \beta_r(t) \sum_{a'} \beta_{p,a,a'} C_{a,a'} \frac{I_{a',r,j}(t) + \tau BTI_{a',r,j}(t)}{P_{a',r}(t)}.$$

The proportion of infectious individuals (last term of the right-hand side) is a model outcome at each time step. In detail, $0 \leq \tau \leq 1$ quantifies the reduced transmissibility of breakthrough infections, i.e., due to vaccine failure. Throughout our analysis, we assume $\tau = 0.55$ as sensitivity analysis on this parameter shows that the overall transmission is not significantly affected by its value. $P_{a,r}(t)$ corresponds to the overall number of individuals at time t by age a and region r and is obtained as the sum of all compartments. Due to their small relative weight, we point out that hospitalized individuals were included in the denominator term as an approximation. The derived infectious proportion of the population is combined with parameters $\{\beta_j, \beta_a, \phi_p, \beta_r(t), \beta_{p,a,a'}, C_{a,a'}\}$, where:

  - β_j represents variant-specific contributions to the FoI. It is the product of baseline value for the wild type $\beta_w = 0.014$ and of the variant-specific improvement in transmissibility; 1.55 times higher for the Alpha variant, 2.54 times higher for the Delta variant (+64% compared to the Alpha variant, in turn) [3]. The optimal value for β_w is estimated based on an alternate grid search procedure.
  - β_a represents the age-class specific reduction in susceptibility. We use $\beta_{0-12} = 1.0$ for children (no reduction), $\beta_{13-19} = 0.68$ for teenagers, and $\beta_a = 0.86$ for other adults. As a remark, other than the purely medical information, we expect such values to capture also the different behavior characterizing age classes.
  - ϕ_p represents mitigation effects on transmission dynamics, as induced by the sets of restrictions adopted within each policy regime of the tier system. Values derive from the retail and recreation mobility indicator in the Google Community Mobility Reports [4]. The indicator exhibits the highest correlation with the values of the reproduction number at the regional level. Mobility reductions range from 4.2% (white zone), to 33% (red zone). We account for policy changes during summer 2021 by considering data in May – June 2021 and mobility levels during summer 2020 when similar rules were in place. Moreover, we temporarily adjust mobility levels to reflect specific events: the large spontaneous celebrations across the country accompanying the European Football Championship at the beginning of the summer (+7%) [5], the loosening of restrictions following the introduction of the EU Digital Covid Certificate in August 2021 (+5%) [6].

- $\beta_r(t)$ summarizes the region- and temperature-specific effects and responsiveness to the mitigation strategies adopted. We take into account the possible contribution of seasonal conditions in reducing virus transmission by introducing a regional-specific correction factor that depends on the deviations of temperatures from the median value over the year (see Further information on model parameters).
- Product term $\beta_{p,a,a'}C_{a,a'}$ represents the average number of effective daily contacts between individuals from age classes a and a', as designed by the Polymod matrix [7]. We exploit the dependence of $\beta_{p,a,a'}$ on tier p to reduce the average number of effective daily contacts among children and teenagers only to account for age-specific provisions adopted on school closures and remote learning, i.e., $\beta_{p,a,a'}$ differs from 1 if $a = a'$ and $a \in \{0-12, 13-19\}$. We obtain the values for the two age groups using data on mobility and school restrictions and validate them throughout the available periods. We set them to 0.3 for the red zone, 0.5 for the orange one, 0.7 for the yellow one, and 0.75 for the white one. Over the summer, we set 0.6 as the baseline and consider values 0.5 and 0.8, respectively, for *Optimistic rollout* and *Pessimistic rollout.*

Beyond natural infection, susceptible people may leave the compartment following the first dose of the vaccine ($d = 1$), according to monthly age-specific coverage rates $\eta_{k,a}(t)$, for vaccine type k. Vaccinated individuals receive a second dose ($d = 2$) after a pre-determined interval, depending on vaccine type (21 days and 90 days, respectively, for $k = 1$ and $k = 2$).

- $\mathbf{V_{k,1}}$: We consider two sets of vaccines. The first set ($k = 1$) consists of Pfizer BioNTech and Moderna vaccines, whereas the second includes the Oxford-AstraZeneca and Johnson & Johnson vaccines. The two vaccine types are administered based on different timing and are targeted to different age groups in line with the government's plan. The two vaccine types also differ in their efficacy. We account for observed regional rollout schedules (see S4 Vaccine rollout and coverage rate scenarios). All vaccines require two doses to reach full effectiveness, with different administration schedules. Since the marginal contribution of the single-dose vaccine (Johnson & Johnson) is negligible – about 3.1% of the fully vaccinated people as of January 31, 2022 – we assume that all vaccines belonging to group 2 require two doses for the sake of simplicity. We model such a setup by accounting for a first compartment, $V_{k,1}$, where susceptible vaccinees are transferred with age-specific coverage rate $\eta_{k,a}$, $k = 1, 2$. Once in the compartment, people may either acquire the disease due to vaccine failure – i.e., the complement to one of vaccine efficacy $\varepsilon_{a,r,k,j,1}(t)$ - or receive the second dose with rates $\sigma_1 = \frac{1}{21 \text{ days}}$ and $\sigma_2 = \frac{1}{90 \text{ days}}$ and move to the respective compartment $V_{k,2}$.
- $\mathbf{V_{k,2}}$: People who received both doses of vaccine type k are exposed to breakthrough infection, net of vaccine efficacy values $\varepsilon_{a,r,k,j,2}(t)$. Param-

eters $\varepsilon_{a,r,k,j,2}(t)$ account for the administration of the booster doses, following the government's plan (see S4 Vaccine rollout and coverage rate scenarios).

- $\mathbf{I_j}$: At each time step, susceptible individuals are exposed to the risk of acquiring the infection and becoming infectious with variant 1, the incumbent variant Alpha, or variant 2, the new variant Delta. Throughout their stay in the infectious compartments, individuals contribute to the respective FoI. They finally move to the Recovered compartment, **R**, following a generation time $\gamma^{-1} = 5.6$ days [8] or are hospitalized in ICU or non-critical medical areas (MA). In the former case, we assume that individuals no longer contribute to the FoI because they either recover from the disease or get tested and isolate themselves until complete recovery. As a technical remark, hospital admissions occur with a systematic delay of around four days in the latter case. Another technical assumption is that people in the infectious compartments are not subject to any death risk from the disease.

- $\mathbf{BTI_j}$, $j = \delta, \alpha$: Vaccinated individuals that acquire the disease due to vaccine failure against virus type Alpha or Delta enter the corresponding infectious compartment, then leave it at a rate of γ. At the regional level, the efficacy of vaccine type k against severe infections from variant j after d doses, i.e., $\varepsilon_{a,r,k,j,d}(t)$, is modeled based on the attained coverage rates for each age group and the vaccination cohorts determined by the timing of administration. The last assumption allows us to account for the waning immunity. For the sake of simplicity, we use constant efficacy values against the severe disease following the first dose, i.e., $\varepsilon_{a,r,k,j,1}(t) = \varepsilon_{k,j,1}$ (see Table S1).

- **MA**, **ICU**, **MA**$_{\textbf{BT}}$, and **ICU**$_{\textbf{BT}}$: People leaving infectious compartments may fully recover or be hospitalized with a two-day delay following self-isolation. Hospitalization entails admission either to the medical area (MA) or intensive care unit (ICU). To model *hospital admissions*, we retain a share $\xi_{j,a}$ of the individuals leaving $\mathbf{I_j}$ at a rate of γ, $j = \delta, \alpha$. $\xi_{j,a}$ represents the variant- and age-specific probability of hospitalization among infected individuals. Hospital admissions into **MA**$_{\textbf{BT}}$ and **ICU**$_{\textbf{BT}}$ occur according to $\xi'_{j,a}$, which is equal to $\xi_{j,a}$ scaled down by a constant average factor of 0.3 $(0.15 - 0.45)$ [3]. Among hospitalized individuals, an age-specific proportion, ι_a, is admitted to intensive care units, i.e., ICUs, whereas the remainder is conveyed to medical areas, i.e. MAs. Values for $\xi_{j,a}$ and ι_a are derived using individual information in the ISS database and increase with age. Hospitalized individuals leave the MA and ICU stages at rates of γ_{MA} and γ_{ICU}, respectively. Among these, an age-specific proportion dies according to an age-varying parameter μ_a that is computed based on the ISS COVID-19 surveillance data on fatalities and hospitalizations.

- **R**: Recovered individuals from the natural disease move into the recovered compartment, where they are no longer susceptible to the virus throughout their stay. We assume that immunity from the disease cannot wane [9], which seems sensible in the context of a single wave. The individuals of the **R** compartment with one of the two sets of vaccines available move into the stage of vaccinated individuals that are no longer susceptible to any SARS-CoV-2 infection denoted as **VR**. This assumption seems reasonable in the context of infections related to Alpha and Delta, in which the overall number of reinfections was negligible. The initial share of recovered individuals in each region is estimated from hospitalizations and fatality rates as described above:

$$R_{a,r}(t_0) = INF_{a,r}(t_0) - \sum_{s}^{t_0} M_{a,r}(s),$$

  where $M_{a,r}(t)$ denotes the observed number of deaths for a and r at time t.

- **VR**: Vaccinated individuals developing breakthrough disease from infection are assumed to leave the **BTI** compartment on average after five days and acquire complete immunity. Unlike the compartment **R** of individuals who recovered from natural infection, subjects in **VR** are no longer eligible for a vaccine by definition.

The model considers two main trajectories: the dynamics of natural infections and that induced by vaccination. For each a and r and given p, model dynamics are described by the following ordinary differential equation system:

$$S'_{a,r}(t) = -\left(\sum_j \lambda_{a,r,j,p}(t) + \sum_k \eta_{k,a}(t)\right) S_{a,r}(t)$$

$$I'_{a,r,j}(t) = \lambda_{a,r,j,p}(t) S_{a,r}(t) - \gamma I_{a,r,j}(t)$$

$$MA'_{a,r}(t) = \gamma(1-\iota_a)\sum_j \xi_{j,a} I_{a,r,j}(t) - \gamma_{MA} MA_{a,r}(t)$$

$$ICU'_{a,r}(t) = \gamma\iota_a \sum_j \xi_{j,a} I_{a,r,j}(t) - \gamma_{ICU} ICU_{a,r}(t)$$

$$R'_{a,r}(t) = \gamma\sum_j (1-\xi_{j,a}) I_{a,r,j}(t) + (1-\mu_a)(\gamma_{MA} MA_{a,r}(t) + \\ + \gamma_{ICU} ICU_{a,r}(t)) - \sum_k \eta_{k,a}(t) R_{a,r}(t)$$

$$V'_{a,r,k,1}(t) = \eta_{k,a}(t) S_{a,r}(t) - (\sigma_k + \sum_j (1-\varepsilon_{a,r,k,j,1}(t))\lambda_{a,r,j,p}(t)) V_{a,r,k,1}(t)$$

$$V'_{a,r,k,2}(t) = \sigma_k V_{a,r,k,1}(t) - \sum_j (1-\varepsilon_{a,r,k,j,2}(t))\lambda_{a,r,j,p}(t) V_{a,r,k,2}(t)$$

$$BTI'_{a,r,j}(t) = \lambda_{a,r,j,p}(t)\sum_k ((1-\varepsilon_{a,r,k,j,1}(t))V_{a,r,k,1}(t) + (1-\varepsilon_{a,r,k,j,2}(t))V_{a,r,k,2}(t)) \\ - \gamma BTI_{a,r,j}(t)$$

$$MA_{BT}{}'_{a,r}(t) = \gamma(1-\iota_a)\sum_j \xi'_{j,a} BTI_{a,r,j}(t) - \gamma_{MA} MA_{BT}{}_{a,r}(t)$$

$$ICU_{BT}{}'_{a,r}(t) = \gamma\iota_a \sum_j \xi'_{j,a} BTI_{a,r,j}(t) - \gamma_{ICU} ICU_{BT}{}_{a,r}(t)$$

$$VR'_{a,r}(t) = \sum_k \eta_{k,a}(t) R_{a,r}(t) + \gamma\sum_j (1-\xi'_{j,a}) BTI_{a,r,j}(t) \\ + (1-\mu_a)(\gamma_{MA} MA_{BT}{}_{a,r}(t) + \gamma_{ICU} ICU_{BT}{}_{a,r}(t))$$

We derive approximate solutions to the model equations using the fourth- and fifth-order Runge-Kutta-Fehlberg solver method with daily step size controls [10]. We choose the solver from the *GSL-ODE* library and implement our system of equations using the C++ language.

Further information on model parameters

This subsection provides further information concerning the derivation of the model parameters described above.

Most model parameters are adopted from published estimates or directly estimated from ISS COVID-19 Surveillance epidemic data [1] (see Table 1 in the Supplementary Information).

The parameters adopted from published estimates are: the generation time, γ^{-1} [8]; a reduction factor for hospitalizations among breakthrough infections, $\xi'_{j,a}$ [3]; age-specific infection fatality rate, IFR_a [2]; vaccine efficacy (dependent on age group, region, vaccine type, variant, vaccine dose and time), $\varepsilon_{a,r,k,j,d}(t)$ [3]. Regarding vaccine efficacy, we rely on Italian Civil Protection data to model waning immunity [11]. The data contain aggregate information on vaccine roll-out by date, region, age group, and vaccine type. We collapse this information by month, age-group, region, and vaccine type. Hence, we can compute the *age* of a vaccinee cohort for each month. Knowing the efficacy of a vaccine at different times from its administration allows us to compute the efficacy for each vintage, region, and age group and to use in the model.

We derive the following group of parameters directly from the data provided by the ISS: age-specific proportion of hospital admissions among infected individuals for variant type j, $\xi_{j,a}$; age-specific share of individuals admitted to ICUs among hospitalized individuals, ι_a; recovery rate of individuals hospitalized in MAs, γ_{MA}; recovery rate of individuals hospitalized in ICUs, γ_{ICU}; age-specific fatalities to hospitalizations ratios, m_a.

We calibrate the two sets of free model parameters for each considered region.

The first set of parameters represents the relative regional effectiveness of tier provisions, $\beta_r(t)$. These parameters adjust the transmissibility to reflect local dynamics relatively to a national baseline.

For each region, we find the value minimizing the mean squared error between actual and model-based incidence through a grid search (with step size $\epsilon = 1e-4$) for the period November 9 – December 30, 2020. The grid derives from regressing the region reproduction number on the local policy regime, a time dummy, and region dummies, between October 1, 2020 – January 25, 2021, with the 95%-confidence interval associated with each regional coefficient providing us with the boundaries of the grid itself (values in Table 1).

We rely on the observed tier restrictions, shutting down the model component reproducing the algorithm for policy tiers. We successively validate the results obtained in the first step by restoring the algorithmic component so that the restrictions are determined endogenously within our model. Estimated regional effects are reported in Table 1, together with the ranges used for the grid search.

Concerning seasonal conditions, the literature finds an inverse relationship between temperatures and SARS-CoV-2 transmission, which may be not only strictly related to the direct effects of higher temperatures on the virus but also to other causes, e.g., more frequent outdoors social interactions with warm weather conditions [12]. Nonetheless, there is no clear consensus on the quantitative effects of temperatures [13]. Based on the available evidence, we assume that an increase of one degree Celsius decreases the regional effects by 0.015. Given the high uncertainty about this effect, we also consider the range of [0.005-0.025]. We obtain daily average temperatures for all Italian regions over the past ten years (downloaded from www.ilmeteo.it). Given the high transmissibility of the Delta variant and the unprecedented outbreaks also observed in the warmest areas of the globe, we assume a low-temperature effect on trans-

missibility (0.005). We also explore the effect of considering alternative values for this parameter in the range of $[0.0025 - 0.0075]$.

The second set of parameters concerns the regional relative prevalence of variant 2, Delta, on June 14, 2021. During the period of our simulations (second half of 2021), Delta was replacing Alpha as the most prevalent variant circulating in the country [14, 15].

We calibrated the regional prevalences of Delta on June 14 by a grid search minimizing the mean squared error between the actual and model-based incidence over three weeks (June 14 – July 4, 2021), taking the restrictions as given. To this extent, we consider a uniform grid between 0 and 1, with the same step size ϵ used for regional effectiveness parameters. As before, we validate our procedure by allowing the restriction tiers to be determined endogenously by the interaction between the epidemic and policy.

Region	Fixed Effects	Delta Prevalence
Piedmont	0.950 [0.998,1.095]	0.964
Aosta Valley	1.092 [1.082,1.178]	0.900
Lombardy	1.236 [1.157,1.254]	0.618
Veneto	1.015 [0.889, 1.187]	0.889
Friuli-Venezia Giulia	0.980 [0.904, 1.001]	0.294
Liguria	1.034 [0.975, 1.072]	0.667
Emilia-Romagna	1.242 [1.046,1.243]	0.768
Tuscany	1.002 [0.926,1.023]	0.930
Umbria	1.001 [0.984, 1.081]	0.900
Marche	0.942 [0.878, 0.975]	0.556
Lazio	0.977 [0.856, 0.984]	0.651
Abruzzo	1.173 [1.088, 1.183]	0.437
Molise	0.888 [0.872, 0.970]	0.900
Campania	0.861 [0.850, 0.946]	0.705
Apulia	1.075 [0.978, 1.075]	0.838
Basilicata	0.885 [0.879, 0.976]	0.901
Calabria	0.909 [0.909, 1.005]	0.700
Sicily	0.985 [0.905, 1.001]	0.971
Sardinia	0.833 [0.779, 0.877]	0.333
Autonomous Province of Bolzano	1.075 [1.072, 1.179]	0.402
Autonomous Province of Trento	1.105 [0.944, 1.242]	0.903

Table 1: Derived values and ranges for fixed regional effects and derived prevalence of the Delta variant for new infections occurring on June 14, 2021. On fixed regional effects, upper and lower bounds represent the extrema of 95%-confidence intervals of the coefficients of regional dummies in a regression of regional reproduction number on time fixed effects and lagged policy regimes (and regional dummies) for the period October 1, 2020 – January 25, 2021. Derived values are combined with seasonal effects. On Delta prevalence, optimal values are derived from the unit interval, to track the regional growth in the number of new infections occurring from June 14 to July 4, 2021.

References

[1] Riccardo F, Ajelli M, Andrianou XD, Bella A, Del Manso M, Fabiani M, et al. Epidemiological characteristics of Covid-19 cases and estimates of the reproductive numbers 1 month into the epidemic, Italy, 28 January to 31 March 2020. Euro Surveill. 2020;25. doi:https://doi.org/10.2807/1560-7917.ES.2020.25.49.2000790.

[2] O'Driscoll M, Ribeiro Dos Santos G, Wang L, Cummings DAT, Azman AS, Paireau J, et al. Age-specific mortality and immunity patterns of SARS-CoV-2. Nature. 2021;590:140–145. doi:https://doi.org/10.1038/s41586-020-2918-0.

[3] Lopez Bernal J, Andrews N, Gower C, Gallagher E, Simmons R, Thelwall S, et al. Effectiveness of Covid-19 Vaccines against the B.1.617.2 (Delta) Variant. N Engl J Med. 2021;385:585–594. doi:https://doi.org/10.1056/NEJMoa2108891.

[4] LLC G. Google Covid-19 Community Mobility Reports; 2022.

[5] Riccardo F, Frisicale EM, Guzzetta G, Ferraro F, Merler S, Maringhini G, et al. Winning during a pandemic: epidemiology of SARS-CoV-2 during EURO2020 in Italy. Epidemiology and Infection. 2022; p. 1–22. doi:https://doi.org/10.1017/S0950268822000723.

[6] Oliu-Barton M, Pradelski B, Woloszko N, Guetta-Jeanrenaud L, Aghion P, Artus P, et al. The effect of Covid certificates on vaccine uptake, health outcomes, and the economy. Nature Communications. 2022;13(3942). doi:https://doi.org/10.1038/s41467-022-31394-1.

[7] Mossong J, Hens N, Jit M, Beutels P, Auranen K, Mikolajczyk R. Social Contacts and Mixing Patterns Relevant to the Spread of Infectious Diseases. PLos Med. 2008;5(3)(: e74). doi:https://doi.org/10.1371/journal.pmed.0050074.

[8] Linton NM, Kobayashi T, Yang Y, Hayashi K, Akhmetzhanov AR, Jung SM, et al. Incubation Period and Other Epidemiological Characteristics of 2019 Novel Coronavirus Infections with Right Truncation: A Statistical Analysis of Publicly Available Case Data. Journal of clinical medicine. 2020;9. doi:https://doi.org/10.3390/jcm9020538.

[9] Dorigatti I, Lavezzo E, Manuto L, Ciavarella C, Pacenti M, Boldrin C, et al. SARS-CoV-2 antibody dynamics and transmission from community-wide serological testing in the Italian municipality of Vo'. Nat Commun. 2021;12(4383). doi:https://doi.org/10.1038/s41467-021-24622-7.

[10] Fehlberg E. Low-order classical Runge-Kutta formulas with stepsize control and their application to some heat transfer problems. vol. 315. National aeronautics and space administration; 1969.

[11] Italian Civil Protection. Covid-19 Opendata Vaccini; 2022.

[12] Majumder P, Ray PP. A systematic review and meta-analysis on correlation of weather with Covid-19. Sci Rep. 2021;11(10746). doi:https://doi.org/10.1038/s41598-021-90300-9.

[13] Byun WS, Heo SW, Jo G, Kim JW, Kim S, Lee S, et al. Is coronavirus disease (Covid-19) seasonal? A critical analysis of empirical and epidemiological studies at global and local scales. Environmental Research. 2021;196(110972). doi:https://doi.org/10.1016/j.envres.2021.110972.

[14] Istituto Superiore di Sanità. Stima della prevalenza delle varianti VOC (Variants of Concern) in Italia: B.1.1.7, B.1.351, P.1 e B.1.617.2, e altre varianti di SARS-CoV-2 (indagine del 22/6/2021); 2021.

[15] Istituto Superiore di Sanità. Stima della prevalenza delle varianti VOC (Variants of Concern) in Italia: B.1.1.7, B.1.351, P.1 e B.1.617.2, e altre varianti di SARS-CoV-2 (Indagine del 20/7/2021); 2021.
