# Supplementary material for: An epidemic model for SARS-CoV-2 with self-adaptive containment measures
Source: PLoS One. 2022 Jul 25;17(7):e0272009. doi: 10.1371/journal.pone.0272009 (PMC9312378; doi:10.1371/journal.pone.0272009)
Supplement: S2 Appendix — (PDF) [file pone.0272009.s002.pdf]

S2 Appendix. The tier system in Italy

This appendix summarizes the tier system in Italy and is based on [1].

The Italian Ministry of Health elaborated two algorithms in April 2020 to evaluate the probability of a spread and its impact (Fig 1).

The ground of the evaluation relied on 21 indicators (Table 1)[2]. The evaluation envisaged four different levels of probability and impact: *i*) very low, *ii*) low, *iii*) moderate, *iv*) high. Their combination implied the risk of each area (Fig 2.

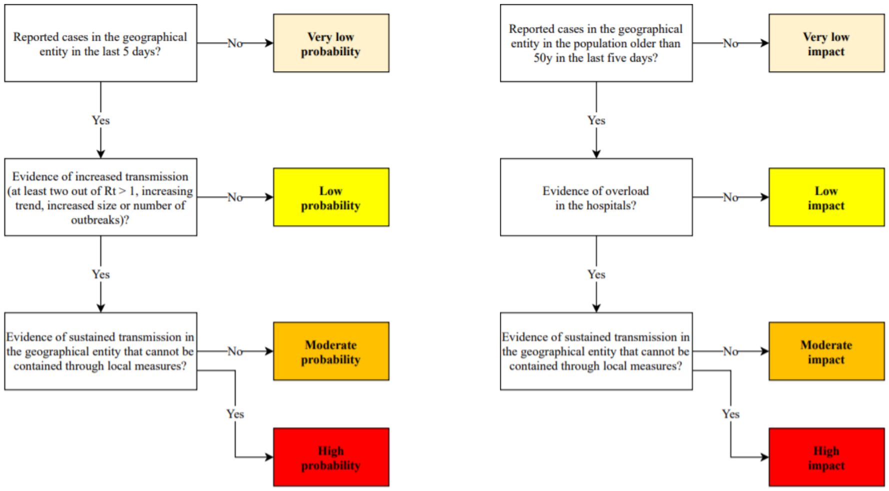

Figure 1: Risk algorithm: probability and impact, Decree Ministry of Health April 30, 2020.

Between November 2020 – March 2022, Italy adopted differential COVID-19 restrictions (zones) at the regional level[3].

Initially, the classification of regions into tiers characterized by different levels of restriction relied on evaluating the epidemic risk (as a combination of probability of epidemic spread and the associated impact) in each area and the level of Rt.

The other layer to define the policy measures in each geographical entity relied on the level of Rt. In August 2020, the Health Minister defined four scenarios depending on the level of Rt. The four scenarios are the following: 1) $Rt < 1$, 2) $1 \leq Rt < 1.25$, 3) $1.25 \leq Rt < 1.5$, 4) $Rt \geq 1.5$.

The Decree November 3, 2020, defined the zones in the following way:

1. High/Very high risk and Scenario 4 → “Red zone”.

2. High/Very high risk and Scenario 3 → “Orange zone”.

3. Any level of risk and Scenario 1 or 2 → “Yellow zone”.

The tier assignment framework underwent several updates over time.

In January 2021, the Italian Minister of Health included an additional indicator based on the incidence in the area and a “white zone” for situations in which both low incidence (i.e., less than 50 cases per 100,000 inhabitants) and risk occur (Fig 2). The Decree February 23, 2021 slightly changed the algorithm (Fig 3). In particular, with low incidence, the yellow zone also applies if the region is at high risk and *Rt* is larger than 1.25 (Scenario 3 or 4). From March, the red zone restrictions also trigger if the weekly incidence exceeds 250 cases per 100,000 inhabitants. This mechanism corresponds to *Rt-New positives* in the main text.

With the decree May 18, 2021, incidence became a crucial parameter. The decree envisaged four different levels of incidence: lower than 50 cases per 100,000 inhabitants, lower than 150 cases, lower than 250 cases, and above 250 cases. Together with the risk, the incidence level defined the zone of a geographical entity (Fig 4. This mechanism corresponds to *Incidence* in the main text.

Between July 23, 2021 – March 31, 2022 (end of the emergency state, initially declared on January 31, 2020, and regional tiers), the new criteria mainly relied on bed occupancy rates in hospitals (see Fig 5). In detail, the white zone applied with a weekly incidence below 50 cases per 100.000 inhabitants or with ICU (resp., hospital bed) occupancy rates below 10% (resp., 15%). When the incidence was between 50 and 150 cases and the criteria for the white zones were not met, the yellow zone applied. Alternatively, it also applied if the incidence was above 150 and ICU occupancy (resp., hospital bed) occupancy rate was below 20% (resp., 30%). When the weekly incidence was above 150 cases and the criteria for the yellow zone were not met, the orange zone applied with ICU and hospital bed occupancy rates above 20% and 30%, respectively. When both rates exceeded 30% and 40%, respectively, and the incidence was above 150 cases inhabitants, the red zone applied. This mechanism corresponds to *Occupancy rates* in the main text.

For each mechanism, the model assignment of the restrictions to a region follows its real-world design closely. However, we neglect long-term care indicators in assessing risk.

Impact \ Probability	**Very low**	**Low**	**Moderate**	**High**
**Very low**	Very low risk	Low risk	Low risk	Moderate risk
**Low**	Low risk	Low risk	Moderate risk	Moderate risk
**Moderate**	Low risk	Moderate risk	Moderate risk	High risk
**High**	Moderate risk	Moderate risk	High risk	Very high risk

Figure 2: Probability-impact matrix, Decree Ministry of Health April 30, 2020.

Area	Indicator number	Indicator
Monitoring:<br>Quality of data collected<br>at the national level	1.1	Percentage of symptomatic cases with date of symptom onset reported
	1.2	Percentage of cases admitted to hospital (non-intensive care ward) with a date of admission or transfer reported
	1.3	Percentage of cases admitted to hospital (intensive care unit) with a date of admission or transfer reported
	1.4	Percentage of notified cases with the municipality of residence reported
Ability to test all cases<br>in a timely manner	2.1	Percentage of swabs positive for SARS-CoV-2 infection per month, excluding swabs from screening and re-testing; overall and by setting (local, non-hospital, hospital emergency department, other)
	2.2	Time between date of symptom onset and date of diagnosis of cases
Adequacy of staff resources<br>for contact tracing, isolation<br>and quarantine	2.4	Number of staff dedicated to contact tracing in each local health unit
	2.5	Number of staff dedicated in each unit to the activities for the collection and dispatch of clinical samples to the reference laboratories and monitoring of cases and close contacts placed in quarantine and in isolation, respectively
	2.6	Number of confirmed cases in the region for which an epidemiological investigation has been carried out, with the search for close and total contacts of new confirmed cases
Transmission<br>stability	3.1	Number of cases diagnosed in the last 14 days, reported to the health ministry
	3.2	Value of transmissibility parameter based on data from the integrated central surveillance system. Two indicators are used, one based on date of symptom onset and one on date of hospitalization
	3.4	Number of cases per date of diagnosis and date of symptom onset reported to the integrated central surveillance system per day
	3.5	Number of new SARS-CoV-2 clusters, defined as two or more epidemiologically linked cases or an unexpected increase in the number of cases at a defined time and place
	3.6	Number of new cases not associated with known chains of transmission
Pressure on health-care<br>system	3.8	Bed occupancy rate (percentage of available active hospital beds occupied by COVID-19 patients) in intensive care units for COVID-19 patients
	3.9	Bed occupancy rate (percentage of available active hospital beds occupied by COVID-19 patients) in non-intensive care wards for COVID-19 patients

Table 1: Indicators used by the Italian Ministry of Health for monitoring the epidemic, Decree Ministry of Health April 30, 2020 [3]. Optional indicators (1.5, 1.6, 2.3, 3.3, and 3.7) are not reported.

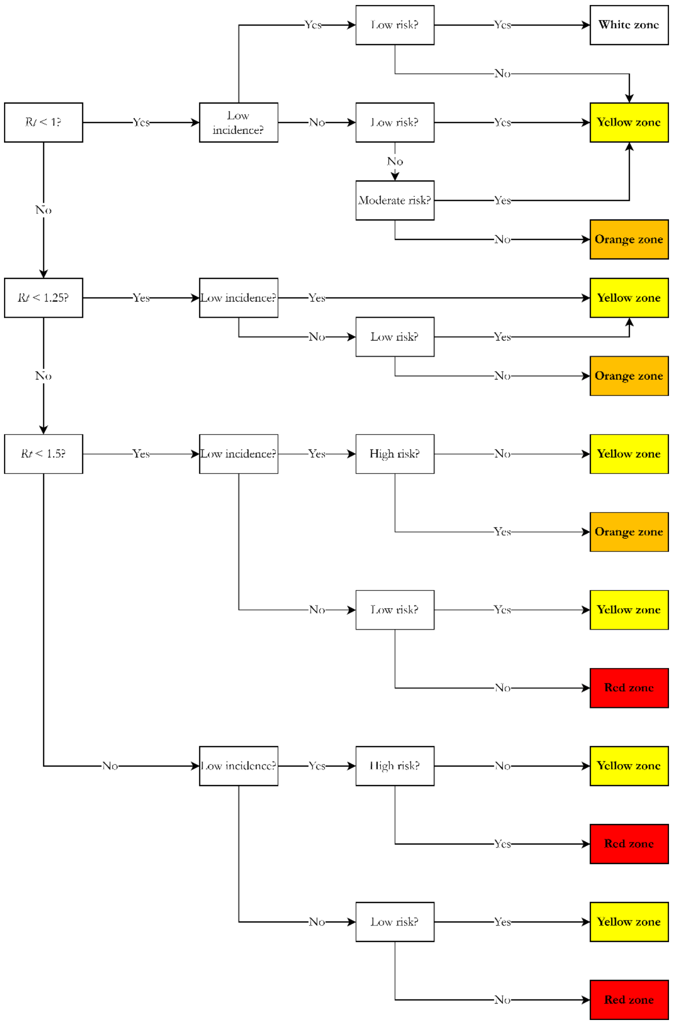

Figure 3: Definition of zones, Decree January, 14 2021. Source: authors' elaboration based on Decree January 14, 2021. The Decree February, 23 2021 slightly changed the algorithm. In particular, when incidence is low, the "Yellow zone" also applies if the region is at high risk and Rt is larger than 1.25 (Scenario 3 or 4).

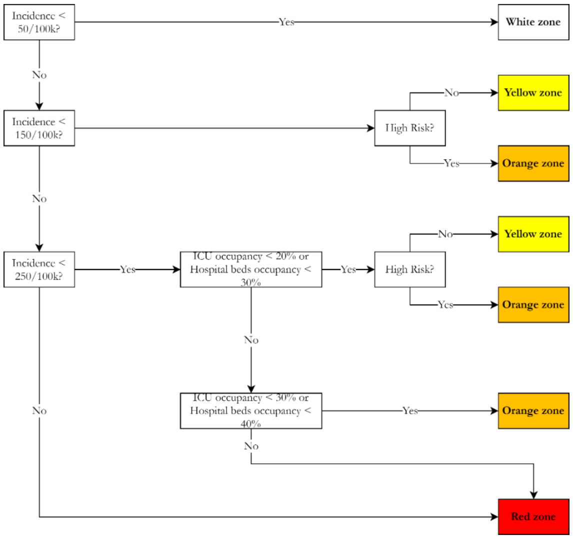

Figure 4: Definition of zones, Decree May 18, 2021.

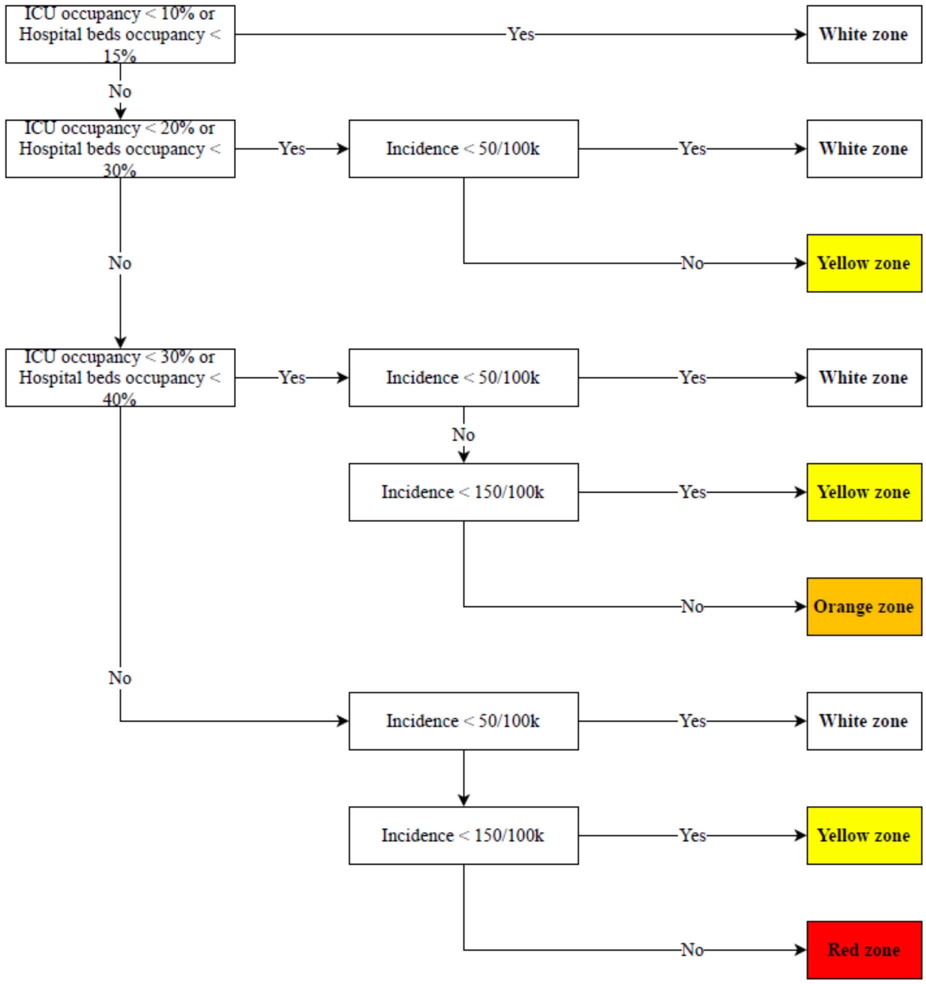

Figure 5: Definition of zones, Decree July 23, 2021.

References

[1] Conteduca FP, Borin A. A new dataset for local and national COVID-19-related restrictions in Italy. The Italian Economic Journal. 2022;8:435–470. doi:https://doi.org/10.1007/s40797-022-00197-0.

[2] Riccardo F, Guzzetta G, Urdiales AM, Del Manso M, Andrianou XD, Bella A. Data for action in Covid-19 response: effectiveness of weekly rapid risk assessments in Italy; 2021.

[3] Riccardo F, Frisicale EM, Guzzetta G, Ferraro F, Merler S, Maringhini G, et al. Winning during a pandemic: epidemiology of SARS-CoV-2 during EURO2020 in Italy. Epidemiology and Infection. 2022; p. 1–22. doi:https://doi.org/10.1017/S0950268822000723.
