# Supplementary material for: An epidemic model for SARS-CoV-2 with self-adaptive containment measures
Source: PLoS One. 2022 Jul 25;17(7):e0272009. doi: 10.1371/journal.pone.0272009 (PMC9312378; doi:10.1371/journal.pone.0272009)
Supplement: S3 Appendix — (PDF) [file pone.0272009.s003.pdf]

S3 The Italian stringency index

This appendix summarizes the construction of the Italian Stringency Index based on [1].

Starting from the Oxford Coronavirus Government Response Tracker[2], we construct a new dataset of the measures enforced at the local level in Italian municipalities, provinces, and regions (in total, more than 7,900 municipalities, 107 provinces, including the two autonomous ones, and 19 regions) starting from January 1, 2020, to March 31, 2022. We rely on local laws available through the official websites of regions and local entities. Moreover, we use the information contained in press articles and releases. We enrich the available information set by including the zone assignment of the regions and autonomous provinces enforced by the central Government via the Ministry of Health. The data allow us to compute the Italian Stringency Index, closely related to the Oxford Stringency Index derived from the Oxford Coronavirus Government Response Tracker.

To account for the intensity and features of the restrictions adopted in Italy (see Table 1), we re-code some of the variables of the Oxford Stringency Index[2]. As in the Oxford Stringency Index, for each region r, we compute a sub-indicator I_j^r of the intensity of the policy measure V_j. In particular, $I_j^r = \frac{v_j^r}{\bar{v}_j^r}$, where v_j^r varies between 0 and $\bar{v}_j^r$, which is the maximum value that V_j can attain. In the expression, we neglect the time index for ease of notation. We also take into account that some measures were in force only on some days of the week (for example, weekend closures of shops in malls selling non-essential goods or services) or assign intermediate values with respect to those reported in Table 1 to account for in-between cases (for example, due to *ad hoc* provisions at the regional level). Finally, we compute an indicator at the regional level by taking the simple average of the nine available sub-indicators. We disaggregate the indicator relative to “Workplace closing” into three sub-indicators: *i*) Production, *ii*) Shops, *iii*) Bars and restaurants. Then, we collapse the three sub-indicators by a simple average.

Variable	Level	Description
C1_School	0	No measures
	0.5	Limited measures
	1	Distance education for upper secondary schools
	1.5	Distance education for upper secondary schools and second and third grade of lower secondary school
	2	Distance education for upper and lower secondary school
	2.5	Distance education for all schools (except for kindergartens)
	3	All school closed or in distance education
C2_1_Production	0	No restrictions
	1	Recommended work from home
	2	Require work from home
	3	Require closing all-but essential workplaces
C2_2_Shops	0	No measures
	1	Limited measures
	2	Limited closures
	3	Ban on non-essential products
C2_3_BarsRestaurants	0	No measures
	1	Dine-in-allowed at some times of the day
	2	Dine-in not-allowed
	3	Require closing
C3_PublicEvents	0	No measures
	1	Limited ban on public events
	2	Ban on all public events

Variable	Level	Description
C4_Gatherings	0	No measures
	1	Restrictions on gatherings above 1,000 people
	2	Restrictions on gatherings up to 1,000 people
	3	Restrictions on gatherings up to 100 people
	4	Restrictions on gatherings up to 10 people
C5_PublicTransport	0	No measures
	1	Limited restrictions
	2	Shutdown of public transport
C6_StayAtHome	0	No measures
	1	Recommended sheltering
	2	Mandatory sheltering (excluded essential activities)
	3	Mandatory sheltering
C7_InternalMovement	0	No measures
	1	Limited measures (e.g. curfew)
	2	No movement between regions
	3	No movement between municipalities
	4	No movement within a municipality
C8_InternationalTravel	0	No measures
	1	Require negative test
	2	Mandatory quarantine
	3	Entry ban on some countries
	4	Entry ban on all countries
H1_PublicCampaigns	0	No measures
	1	Public campaign on some media
	2	Coordinated campaigns on all media

Table 1: Description of the variables used to construct the *Italy Stringency Index*.

References

[1] Conteduca FP, Borin A. A new dataset for local and national COVID-19-related restrictions in Italy. The Italian Economic Journal. 2022;8:435–470. doi:https://doi.org/10.1007/s40797-022-00197-0.

[2] Hale T, Angrist N, Goldszmidt R, Kira B, Petherick A, Webster S, et al. A global panel database of pandemic policies (Oxford Covid-19 Government Response Tracker). Nat Hum Behav. 2021;5:529–538. doi:https://doi.org/10.1038/s41562-021-01079-8.
