# Supplementary material for: An epidemic model for SARS-CoV-2 with self-adaptive containment measures
Source: PLoS One. 2022 Jul 25;17(7):e0272009. doi: 10.1371/journal.pone.0272009 (PMC9312378; doi:10.1371/journal.pone.0272009)
Supplement: S4 Appendix — (PDF) [file pone.0272009.s004.pdf]

S4 Appendix. Vaccine rollout and coverage rate scenarios

This appendix describes the construction of the different vaccination scenarios used in the main text (*Actual rollout*, *Pessimistic rollout*, and *Optimistic rollout*).

Administration rates were determined considering the deliveries of new doses, the administration constraints, the required interval between doses, and historical data (up to December 2021)[1].

For the period December 2021 -– March 2022, we need to make assumptions on the pace of vaccination and booster administration. In particular, in the *Actual rollout*, we assume that only type-1 vaccines are administered to the population since December, in line with the available evidence at the end of November (the share of type-2 vaccines was negligible). We assume an increase in first-dose administration for the last three age groups in December 2021 to fit the fact that accessing the workplace requires either vaccination or a previous infection. In the first quarter of 2022, the vaccination campaign continues at a slower pace. We also assume that the vaccination in the youngest cohort takes off in December 2021 and decreases over time in the first quarter of 2022. As far as it concerns the booster administration, we follow the provisions of the Italian Ministry of Health. At the end of September, a booster was recommended for individuals with a high risk of severe disease, individuals above 80 years old, healthcare workers above 60 years old, or at a high risk of severe disease six months after full vaccination. In October, a booster was also recommended for individuals above 60 and all high-risk individuals. In November and December, the booster was extended to individuals above 40 and 18, respectively. Moreover, people could get the booster five months after completing the vaccination course. Finally, in January, individuals above 12 could receive the booster four months after completing the vaccination course. Besides the *Actual rollout*, we also consider an optimistic scenario (*Optimistic rollout*) and a pessimistic one (*Pessimistic rollout*). *Optimistic rollout* assumes a faster rollout than observed between June and November (20% faster in the second and third age groups, 55% for the fourth age group, and 105% faster for the fifth age group). *Pessimistic rollout* assumes a slower rollout than observed (80% of actual doses for all age groups between June and December). *Pessimistic* (*Optimistic*) *rollout* shows a faster (slower) reduction of first doses than *Actual rollout* from December 2021 to March 2022. *Optimistic* (*Pessimistic*) *rollout* assumes a fast (slow) deployment of boosters to the eligible individuals. Moreover, vaccinated individuals may access boosters four months after the second dose in *Optimistic rollout.* Overall, the allocation of boosters works according to a first-in-first-out principle since we do not have individual-level information on the time span between the vaccinations. In other words, the oldest vaccinees, yet to receive the booster, are the first in line to receive it. Table 1 reports the average coverage by age group and scenario at the end of the period (March 2022).

**Age Group**	***Actual rollout***	***Optimistic rollout***	***Pessimistic rollout***
0-12	39.5%	55.0%	20.7%
13-18	77.8%	90.5%	64.9%
19-64	89.7%	96.8%	80.2%
65-79	97.5%	99.0%	91.0%
80+	98.3%	99.4%	95.0%

Table 1: Average coverage by age group and scenario by March 2022. *Actual rollout* uses historical data (up to December 2021)[1]. *Pessimistic rollout* assumes a slower rollout than actual one. *Optimistic rollout* assumes a faster rollout than actual one.

References

[1] Italian Civil Protection. Covid-19 Opendata Vaccini; 2022.
