# Supplementary material for: An epidemic model for SARS-CoV-2 with self-adaptive containment measures
Source: PLoS One. 2022 Jul 25;17(7):e0272009. doi: 10.1371/journal.pone.0272009 (PMC9312378; doi:10.1371/journal.pone.0272009)
Supplement: S5 Appendix — (PDF) [file pone.0272009.s005.pdf]

# S5 Appendix. Fixed Restrictions vs. self-adaptive rule-based mechanism

Figure 1 displays the counterfactual scenarios in which yellow-, orange-, and red-zone restrictions apply nationwide during the entire simulation period. In this manner, we can compare the results of our modeling framework with those of an epidemiological model that does not embed endogenous adjustments to the containment measures. Figure 1 shows the projections for new cases (top panel) and Italian Stringency Index (ItSI, bottom panel) associated with each fixed policy regime ("yellow", "orange", and "red"), observed and simulated tiers, together with real data on incidence (in the top panel). As one may expect, we observe that the severity and the length of the epidemic wave are inversely related to the restrictiveness of the policies (1, top panel). While a nationwide red zone allows a rapid suppression of the epidemic by the end of the spring, the yellow zone determines a substantial increase of cases until April and a slow reduction until May. The lack of an endogenous adjustment policy mechanism clearly translates into a large epidemic burden (the yellow-zone policy) or disproportionate restrictions (the red-zone policy). Moreover, constant policies do not reflect dynamically the progress of the vaccination campaign, which allows the policymakers to reduce restrictions and the epidemic burden at the same time. The level of restrictions associated with a non-reactive application of red and orange zones would be much larger than the self-adaptive policy mechanism described in this paper (1, bottom panel). On the contrary, the constant application of the yellow zone would result in too lenient restrictions.

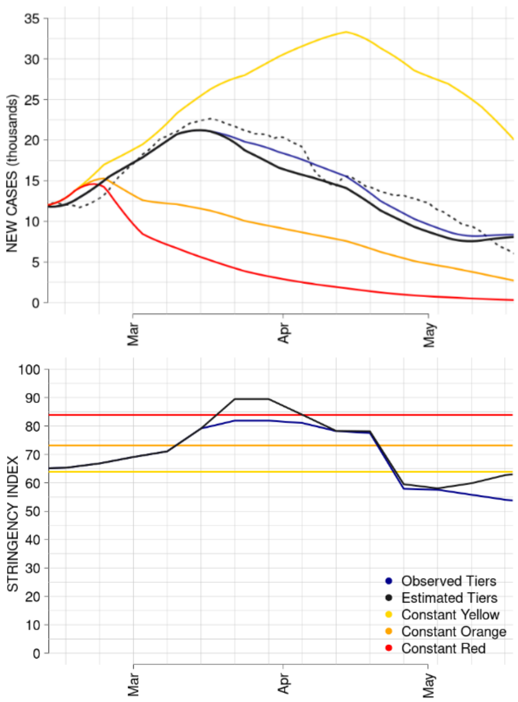


Figure 1: New cases and the Italian Stringency Index (ItSI) during the third wave (March-May 2021), observed, simulated, and with non-responsive nationwide yellow, orange, and red zones. The dashed curve in the top panel reports the observed new cases.
