# Supplementary material for: An epidemic model for SARS-CoV-2 with self-adaptive containment measures
Source: PLoS One. 2022 Jul 25;17(7):e0272009. doi: 10.1371/journal.pone.0272009 (PMC9312378; doi:10.1371/journal.pone.0272009)
Supplement: S6 Appendix — (PDF) [file pone.0272009.s006.pdf]

S6 Appendix. Assessing the "decoupling" between the United States and Italy

This appendix provides an analysis of the potential reasons behind the epidemiological decoupling between the United States and Italy observed at the beginning of 2021.

Since the beginning of 2021, the pandemic has shown heterogeneous trends worldwide: while Europe was facing its third wave, in the United States, infections sharply declined, remaining at relatively lower levels until the end of May, despite enforcing less severe restrictions as compared to Europe. In the first quarter of 2021, the Oxford Stringency Index[1] was 15 points lower in the United States than in Italy and Germany. As measured by the Google COVID-19 Community Mobility Reports,[2] retail and recreation mobility had been gradually growing and approaching pre-pandemic levels by the end of the quarter in the United States, while in Europe, it remained 30%-60% below pre-pandemic levels (see Figure 1).

The different progress of the vaccination campaign has been commonly considered the main factor behind the observed divergence between epidemic trends in the two regions. Indeed, at the end of the first quarter, in the United States, about 30% of the population had received at least one dose of the vaccine compared to just over 11% in Italy (see Figure 2). In spring, the vaccine rollout accelerated in Europe so that the vaccine gap between the United States and Italy was almost entirely closed by mid-June. Besides vaccine rollout, at least one other factor could have played an essential role in favoring the decoupling: the different spread of Alpha, i.e., a more transmissible SARS-CoV-2 variant than the wild type. According to the US Centers for Disease and Control and Prevention, the proportion of US cases attributed to Alpha was around 25%-30% in mid-March compared to 87% in Italy[3, ?]. Other things being equal, this would entail a delay of about 5-6 weeks compared to the progress of the same variant in Italy and most other EU countries. It is hard to evaluate the role of different factors just by looking at the evolution of epidemic and restriction dynamics across countries. Many confounding factors such as initial epidemic conditions, demographic characteristics, differences in containment policies, and individual behaviors may undermine the possibility of getting a precise evaluation of the contribution of a specific component. By considering the endogenous response of containment policies, our modeling framework allows us to analyze how a specific external factor may affect contagion and restrictions once applied to a specific country (Italy in our case).

To provide a quantitative assessment of how the vaccination progress and the initial prevalence of more transmissible variants may have influenced the divergence between Italy and the United States, we construct two *ad hoc* counterfactual scenarios for February-May 2021. We apply to Italy the more favorable conditions prevailing in the United States regarding the vaccine rollout and the prevalence of more transmissible variants. Clearly, there are other possible sources of divergence, such as the possible heterogeneity in initial incidence or

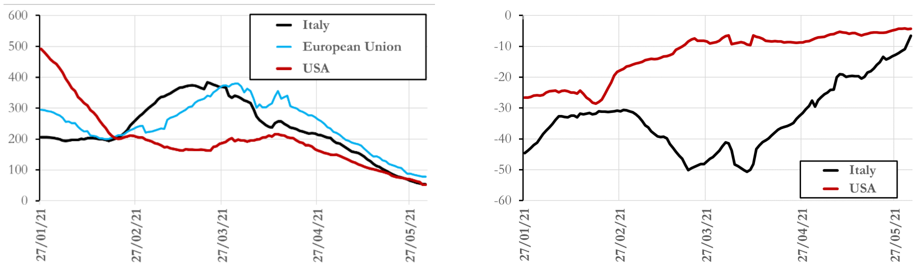

Figure 1: Epidemic and mobility in the US and Italy[4, 2].

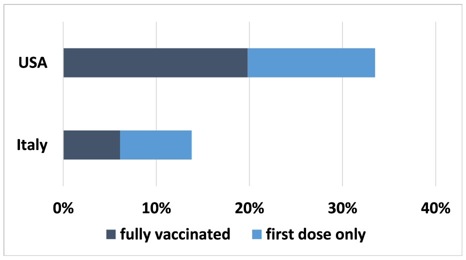

Figure 2: Share of people who received at least one dose of COVID-19 vaccine in the United States and Italy. Source: Our World in Data.

immunity due to previous infections. However, we aim to assess how - *ceteris paribus* - the changes in these two specific conditions (vaccination and prevalence of Alpha) would affect epidemic and stringency conditions in Italy. In the first counterfactual scenario, we assume the spread of Alpha to occur in Italy with a delay of 40 days; in the second one, on top of a delayed diffusion of the variant, we consider a vaccine rollout similar to the one in the United States (see Figure 3).

We examine the evolution of the number of notified cases and the modified version of the Oxford Stringency Index in these two alternative scenarios, comparing them with the realized dynamics (Figure 4). As for the latter, we consider the values produced by simulations instead of actual data so that any difference in the counterfactual scenarios can be attributed only to the variation of external conditions instead of model deviations from actual data. Nevertheless, results would not substantially change when using realized data, given the model's ability to track actual evolution over the considered period (see Figure 7 in Section 4).

The simulations suggest that the main driver for the divergence in February-March was the early diffusion of Alpha in Italy, which is associated with an average increase of 72.5% in notified cases and 8.5 points in the Italian Stringency Index (compare the dashed and brown lines in Figure 4). After realigning the spread of Alpha, a faster vaccination campaign becomes the leading factor (compare the brown and purple lines). In particular, a rapid vaccine rollout

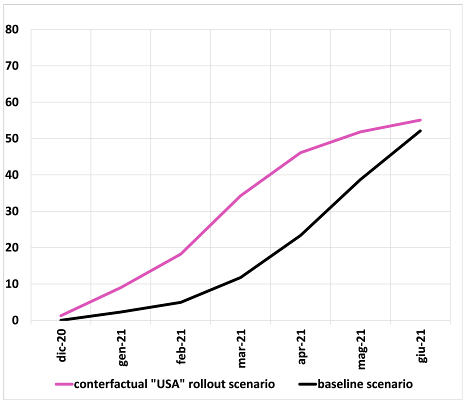

Figure 3: Vaccine rollout scenarios: share of total population covered.

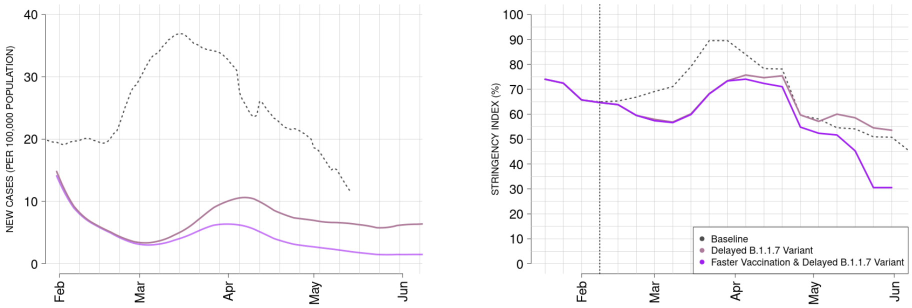

Figure 4: Simulated dynamic of the Italian Stringency Index: counterfactual scenarios.

leads to an average reduction of 56.5% in notified cases and of 7.7 points in the Italian Stringency Index in April–May. Considering the entire simulation period, a delayed diffusion of the new variant reduces by about 60.0% new cases and by 8 points the Italian Stringency Index. A faster vaccine rollout would allow a further relaxation of the policies by additional 3.6 points on average, while the number of cases would have fallen by an additional 13.3%. These results highlight the importance of preventing or delaying the spread of insidious virus variants.

References

[1] Hale T, Angrist N, Goldszmidt R, Kira B, Petherick A, Webster S, et al. A global panel database of pandemic policies (Oxford Covid-19 Government Response Tracker). Nat Hum Behav. 2021;5:529–538. doi:https://doi.org/10.1038/s41562-021-01079-8.

[2] LLC G. Google Covid-19 Community Mobility Reports; 2022.

[3] for Disease Control C, Prevention. Variant Proportions; 2021.

[4] Ritchie H, Mathieu E, Rodés-Guirao L, Appel C, Giattino C, Ortiz-Ospina E, et al.. Coronavirus Pandemic (Covid-19); 2022.
