# Supplementary material for: An epidemic model for SARS-CoV-2 with self-adaptive containment measures
Source: PLoS One. 2022 Jul 25;17(7):e0272009. doi: 10.1371/journal.pone.0272009 (PMC9312378; doi:10.1371/journal.pone.0272009)
Supplement: S1 Fig — (PDF) [file pone.0272009.s007.pdf]

# S1 Figure. Model calibration.

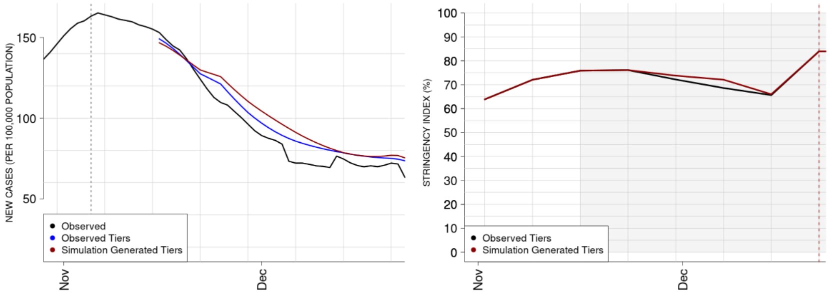


Figure 1: Left panel: incidence. Right panel: Italian Stringency Index (ItSI). *Observed* denotes the actual path of incidence (resp., ItSI) for the period November 9 – December 30, 2020. *Observed Tiers* denotes the simulated path of incidence (resp., ItSI) for the period November 9 – December 30, 2020 under the observed regional tiers. *Simulated Generated Tiers* denotes the simulated path of incidence (resp., ItSI) for the period November 9 – December 30, 2020 when regional tiers are generated endogenously within the model.
