# Supplementary material for: An epidemic model for SARS-CoV-2 with self-adaptive containment measures
Source: PLoS One. 2022 Jul 25;17(7):e0272009. doi: 10.1371/journal.pone.0272009 (PMC9312378; doi:10.1371/journal.pone.0272009)
Supplement: S1 Table — (PDF) [file pone.0272009.s008.pdf]

S1 Table. Model parameters.

PARAMETER	VALUE [min, max]	NOTE
γ^{-1} (generation time, days)	5.6	[1]
$\xi_{w,a}$ (age-specific proportion of hospital admissions among infected individuals for variant type 1)	0-12: 0.004<br>13-18: 0.004<br>19-64: 0.019<br>65-79: 0.062<br>80+: 0.119	Own estimates from the ISS COVID-19 Surveillance data [2]
$\xi_{2,a}$ (age-specific proportion of hospital admissions among infected individuals for variant type 2)	0-12: 0.006<br>13-18: 0.007<br>19-64: 0.031<br>65-79: 0.102<br>80+: 0.194	Own estimates from the ISS COVID-19 Surveillance data[2]
Reduction factor for hospitalizations among breakthrough infections	0.3 [0.15, 0.45]	[3]
ι_a (proportion of ICU admission among hospitalized)	0-12: 0.041<br>13-18: 0.053<br>19-64: 0.129<br>65-79: 0.204<br>80+: 0.104	Own estimates from the ISS COVID-19 Surveillance data[2]
γ_{MA} (MA recovery rate)	0.067	Own estimates from the ISS COVID-19 Surveillance data[2]
γ_{ICU} (ICU recovery rate)	0.056	Own estimates from the ISS COVID-19 Surveillance data[2]
γ_{IFR}	0-12: 0.000016<br>13-18: 0.00002<br>19-64: 0.0014<br>65-79: 0.0198<br>80+: 0.083	[4]
α_a (age-specific fatalities/hospitalizations ratio, national averages)	0-12: 0.004<br>13-18: 0.006<br>19-64: 0.075<br>65-79: 0.315<br>80+: 0.684	Own estimates from the ISS COVID-19 Surveillance data[2]
First dose efficacy of vaccine group 1 for Alpha variant type	0.38 [0.29, 0.45]	[3]
First dose efficacy of vaccine group 2 for Alpha variant type	0.37 [0.32, 0.42]	[3]
First dose efficacy of vaccine group 1 for Delta variant type	0.3 [0.17, 0.41]	[3]
First dose efficacy of vaccine group 2 for Delta variant type	0.18 [0.09, 0.25]	[3]

Table 1: Parameter values. We report the age-specific parameter when applicable.

References

[1] Linton NM, Kobayashi T, Yang Y, Hayashi K, Akhmetzhanov AR, Jung SM, et al. Incubation Period and Other Epidemiological Characteristics of 2019 Novel Coronavirus Infections with Right Truncation: A Statistical Analysis of Publicly Available Case Data. Journal of clinical medicine. 2020;9. doi:https://doi.org/10.3390/jcm9020538.

[2] Riccardo F, Ajelli M, Andrianou XD, Bella A, Del Manso M, Fabiani M, et al. Epidemiological characteristics of Covid-19 cases and estimates of the reproductive numbers 1 month into the epidemic, Italy, 28 January to 31 March 2020. Euro Surveill. 2020;25. doi:https://doi.org/10.2807/1560-7917.ES.2020.25.49.2000790.

[3] Lopez Bernal J, Andrews N, Gower C, Gallagher E, Simmons R, Thelwall S, et al. Effectiveness of Covid-19 Vaccines against the B.1.617.2 (Delta) Variant. N Engl J Med. 2021;385:585–594. doi:https://doi.org/10.1056/NEJMoa2108891.

[4] O'Driscoll M, Ribeiro Dos Santos G, Wang L, Cummings DAT, Azman AS, Paireau J, et al. Age-specific mortality and immunity patterns of SARS-CoV-2. Nature. 2021;590:140–145. doi:https://doi.org/10.1038/s41586-020-2918-0.
