# Supplementary material for: An epidemic model for SARS-CoV-2 with self-adaptive containment measures
Source: PLoS One. 2022 Jul 25;17(7):e0272009. doi: 10.1371/journal.pone.0272009 (PMC9312378; doi:10.1371/journal.pone.0272009)
Supplement: S2 Table — (PDF) [file pone.0272009.s009.pdf]

S2 Table. New positives, MA occupancy rate, ICU occupancy rate, and the ItSI for each mechanism.

Table 1: **Average values of new positives, MA occupancy rate, ICU occupancy rate, and the ItSI for each mechanism.**

Policy mechanism	Daily cases	MA	ICU	ItSI
*Rt-New positives**	2384 [2187, 2965]	3.6% [3.5%, 4.0%]	2.0% [1.9%, 2.5%]	34.3 [33.6, 35.6]
*Incidence**	3836 [3327, 5463]	5.2% [4.7%, 6.6%]	3.3% [2.8%, 4.8%]	28.0 [26.8, 29.8]
*Occupancy rates**	7175 [6331, 8417]	7.8% [7.1%, 8.8%]	5.4% [4.7%, 6.6%]	23.9 [22.7, 26.3]
*Rt-Hospital adm.**	3061 [2748, 3717]	4.5% [4.2%, 5.1%]	2.7% [2.4%, 3.3%]	31.2 [29.9, 34.1]
*Rt-New positives***	2074 [1830, 2202]	3.1% [2.9%, 3.2%]	1.7% [1.6%, 1.9%]	29.2 [26.9, 33.4]
*Incidence***	2809 [2380, 3674]	3.9% [3.6%, 4.6%]	2.4% [2.0%, 3.1%]	24.9 [22.9, 26.9]
*Occupancy rates***	4629 [3315, 6405]	5.3% [4.4%, 6.7%]	3.5% [2.6%, 4.8%]	20.8 [20.3, 22.9]
*Rt-Hospital adm.***	2335 [2071, 2811]	3.5% [3.3%, 3.9%]	2.0% [1.8%, 2.5%]	28.0 [25.5, 30.0]
*Rt-New positives****	3156 [2808, 3837]	4.5% [4.2%, 5.0%]	2.5% [2.3%, 3.1%]	36.4 [35.0, 39.6]
*Incidence****	5327 [4625, 7305]	6.9% [6.3%, 8.4%]	4.4% [3.7%, 5.9%]	30.7 [29.1, 32.0]
*Occupancy rates****	9177 [8553, 10416]	9.8% [9.3%, 10.8%]	6.6% [6.0%, 7.9%]	26.0 [25.2, 28.2]
*Rt-Hospital adm.****	3878 [3558, 4538]	5.5% [5.2%, 6.3%]	3.3% [2.9%, 3.9%]	34.8 [32.4, 39.0]

Each mechanism (*Rt-New positives, Incidence, Occupancy rates, Rt-Hospital admissions*) is considered for the period 07/2021 – 03/2022 and different assumptions regarding vaccine rollout. * indicates *Actual rollout.* ** indicates *Optimistic rollout.* *** indicates *Pessimistic rollout.* Bounds on the scenarios obtained from the sensitivity analysis run on the efficacy of vaccines over time.
